# HIV-1 latency is established preferentially in minimally activated and non-dividing cells during productive infection of primary CD4 T cells

**DOI:** 10.1371/journal.pone.0271674

**Published:** 2022-07-27

**Authors:** Paula C. Soto, Valeri H. Terry, Mary K. Lewinski, Savitha Deshmukh, Nadejda Beliakova-Bethell, Celsa A. Spina

**Affiliations:** 1 Veterans Affairs San Diego Healthcare System, San Diego, California, United States of America; 2 Department of Pathology, University of California San Diego, La Jolla, California, United States of America; 3 Department of Medicine, University of California San Diego, La Jolla, California, United States of America; 4 Veterans Medical Research Foundation, San Diego, California, United States of America; New York University College of Dentistry, UNITED STATES

## Abstract

Latently infected CD4 T cells form a stable reservoir of HIV that leads to life-long viral persistence; the mechanisms involved in establishment of this latency are not well understood. Three scenarios have been proposed: 1) an activated, proliferating cell becomes infected and reverts back to a resting state; 2) an activated cell becomes infected during its return to resting; or 3) infection is established directly in a resting cell. The aim of this study was, therefore, to investigate the relationship between T cell activation and proliferation and the establishment of HIV latency. Isolated primary CD4 cells were infected at different time points before or after TCR-induced stimulation. Cell proliferation within acutely infected cultures was tracked using CFSE viable dye over 14 days; and cell subsets that underwent varying degrees of proliferation were isolated at end of culture by flow cytometric sorting. Recovered cell subpopulations were analyzed for the amount of integrated HIV DNA, and the ability to produce virus, upon a second round of cell stimulation. We show that cell cultures exposed to virus, prior to stimulus addition, contained the highest levels of integrated and replication-competent provirus after returning to quiescence; whereas, cells infected during the height of cell proliferation retained the least. Cells that did not divide or exhibited limited division, following virus exposure and stimulation contained greater amounts of integrated and inducible HIV than did cells that had divided many times. Based on these results, co-culture experiments were conducted to demonstrate that latent infection could be established directly in non-dividing cells via cell-to-cell transmission from autologous productively infected cells. Together, the findings from our studies implicate the likely importance of direct infection of sub-optimally activated T cells in establishment of latently infected reservoirs *in vivo*, especially in CD4 lymphocytes that surround productive viral foci within immune tissue microenvironments.

## Introduction

The latent, stable reservoir of HIV is established early during viral infection in resting CD4 T cells with a predominant memory phenotype [1–5]. Even though the frequency of such latently infected cells in individuals receiving effective antiretroviral therapy is extremely low, the reservoir is long-lived, and eradication is not achieved by inhibiting viral replication alone [6–8]. Research during the past twenty-five years has made considerable advances in understanding the mechanisms of latency establishment [9]. The importance of the cell state at the time of reservoir establishment has become more and more appreciated for potential curative interventions [10, 11]. However, the preferential state of cells which facilitates establishment of latency is still not fully delineated.

Early experiments demonstrated that activated CD4 T cells were dramatically more susceptible to productive HIV infection. It was therefore assumed that the majority of the latent reservoir was formed initially in such productively infected cells that subsequently returned to quiescence. However, it was also well appreciated that activated and dividing T cells with productive infection decay rapidly [12–14] and are unlikely to make a considerable contribution to a long-lived reservoir. To reconcile these conflicting facts, an alternative proposed mechanism has become the most widely accepted one: the best cellular conditions for establishment of latency occur during infection of activated immune T cells, which are in the process of reverting to a resting memory state [15, 16].

It was believed for quite some time that HIV infection could not be established directly in resting primary CD4 T cells. Initial evidence that direct HIV infection of quiescent T cells may occur in lymphoid tissue [17, 18] was not considered definitive, because the results were based on antibody detection of viral proteins *ex vivo*. Presence of HIV detected with this approach could have reflected bound virions, released from infected activated T cells during culture. Using fluorescence in situ hybridization, presence of HIV RNA in phenotypically resting CD4 T cells was detected also, albeit at lower levels compared to activated cells, in lymph node biopsies of people with HIV during acute and early infection [19]. Although considered convincing evidence by many researchers at the time, later mechanistic studies called these results into question, as it did not formally exclude the possibility that HIV replication could arise from unintegrated DNA species [20]. Early *in vitro* studies demonstrated that resting CD4 T cells could be infected with HIV, and reverse transcription proceed to DNA, albeit with delayed kinetics [21, 22]. However, no evidence was given that the reverse transcribed viral genome was integrated. More detailed molecular analyses conducted later by Swiggard et al. [23] demonstrated that resting T cells were almost as susceptible to HIV integration as activated T cells during a single round of infection, although at a much slower pace. These early reports were confirmed subsequently by multiple studies, demonstrating the establishment of latency in resting CD4 T cells via infection by cell-free virus particles [10, 24–26] as well as cell-to-cell transmission [27].

The minimal specific requirements for establishment of HIV infection in primary CD4 T cells have not yet been defined, and the precise level of cell activation that is needed to allow integration of viral DNA remains unknown. Previous reports have shown that HIV vector constructs can infect and integrate into the DNA of non-dividing T cells, following treatment with cytokines present in conditioned medium derived from mitogen-stimulated T cells [28], and that direct infection of resting CD4 T cells can be enhanced following exposure to specific chemokines [24]. Importantly, early exposure to common gamma chain cytokines, such as IL-7 and IL-4, can enhance HIV infection of resting CD4 T cells by blocking rapid induction of apoptosis by viral Vpr and reverse transcription [29]. In addition, studies using dual reporter viruses have demonstrated that chemokine treated resting CD4 T cells can support both productive and latent infection [30] and that the majority of integrated virus becomes latent shortly after infection [31]. Moreover, production of HIV mRNA transcripts and proteins has been detected in phenotypically resting CD4 T cells, either cultured alone [22, 26] or within tonsillar tissue explants [18].

The main goal of our study was to investigate the relationship between T cell activation and the proliferation cycle stage and the establishment of HIV latency in CD4 T lymphocytes. Using primary CD4 T cells *in vitro*, we show that HIV establishes an inducible latent state during acute productive infection, preferentially in minimally activated non-dividing T cells, rather than in fully activated proliferating cells.

## Results

### Design and cell characteristics of the *in vitro* cell culture model

**Fig 1:**
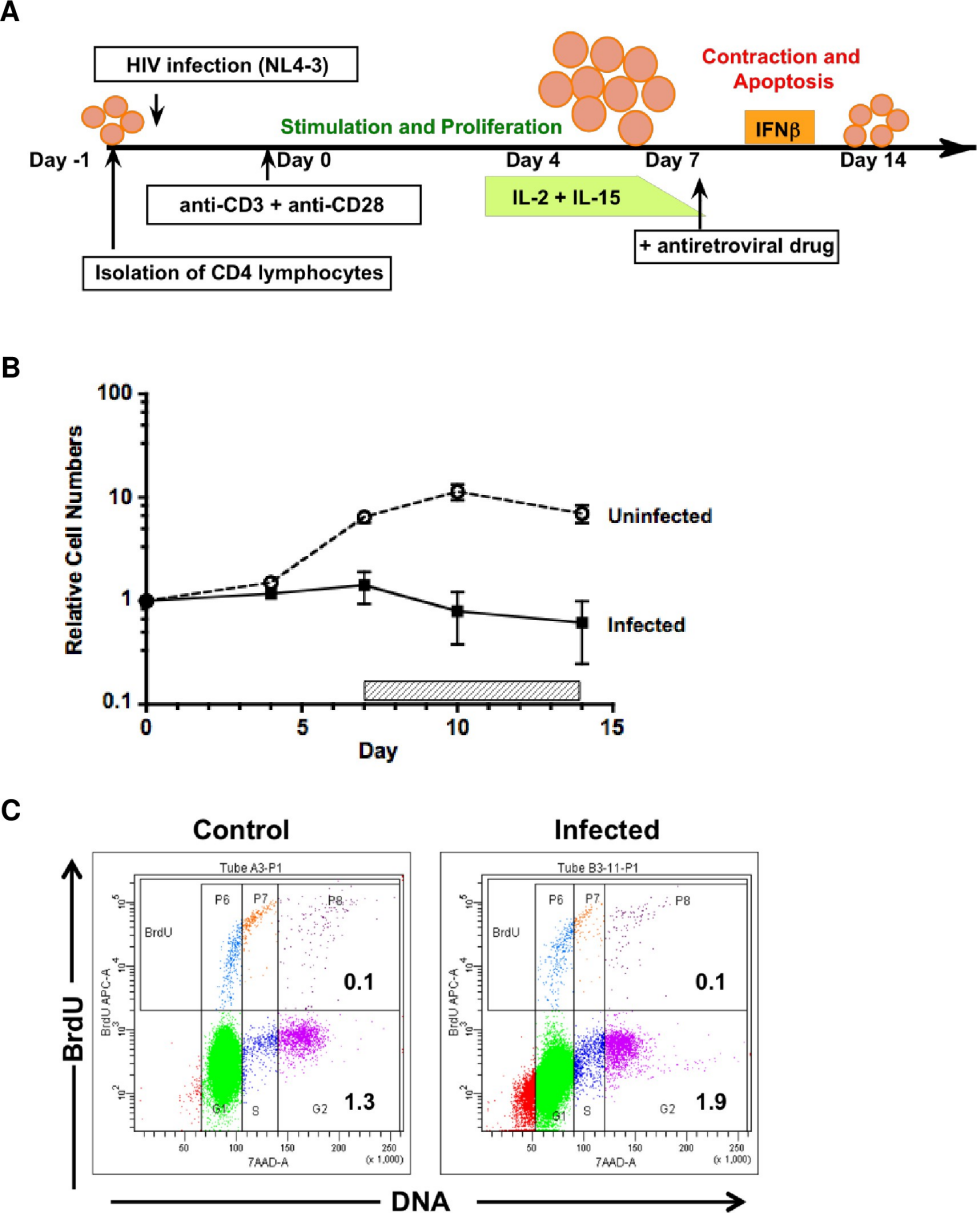
Design and cell characteristics of *in vitro* culture model. (A) Schematic of primary CD4 T cell culture system. Primary CD4 T cells were isolated by negative selection, infected with the NL4-3 clone of HIV-1, and stimulated through TCR cross-linking with plate-bound immobilized anti-CD3 + anti-CD28 antibodies. After 4 days incubation, cells were recovered and transferred to new culture wells that lacked TCR stimulus. During the expansion phase of culture, cells were maintained in medium supplemented with exogenous cytokines, to enhance proliferation. IL-2 was added on day 4; and IL-15 was added on days 4 and 7. During the contraction phase of culture, interferon-beta (IFNβ) was added to the medium on days 10 and 12, to reduce the level of activation-induced cell death. Antiretroviral drugs were added (Nevirapin, at day 5 or Indinavir, at day 7) to prevent further spread of viral infection (refer to details in Materials and Methods for rationale in drug timing). Resting, infected cells were obtained at the end of culture on day 14. (B) Isolated CD4 T cells were either infected with NL4-3 (MOI = 0.01 TCID_50_) or mock infected (Uninfected) under similar experimental conditions and cultured in parallel for 14 days, according to the schema depicted in A. Indinavir was added on day 7 and replenished every two days until the end of culture (horizontal hatched bar). Cell number and viability were measured by trypan blue dye exclusion, along with culture volume, throughout the culture period to calculate the total number of viable cells present at each indicated time point (Days 4, 7, 10, 14). Relative cell numbers were derived by normalization to the initial cell count for each culture condition (infected vs. uninfected control). Results show the mean ± SEM (error bars) from 5 independent experiments, using cells from different donors. Refer to Materials and Methods for complete details of the culture system. (C) Flow cytometry analysis of cell cycle status (7-AAD staining of DNA content) and level of active DNA synthesis (bromodeoxyuridine uptake, BrdU) was performed on aliquots of infected and uninfected CD4 T cells, taken 14 days after culture initiation. For maximal sensitivity, cells were allowed to incorporate BrdU overnight, prior to fixation, permeabilization, and staining. Depiction of events in each cell cycle phase, based on DNA content, has been enhanced by coloration: G_0/1_, green; S, dark blue; G_2_/M, fuchsia. The presence of fragmented DNA (<G_0/1_), found with apoptosis is shown in red. For comparison between the uninfected control and infected cultures, the percentage of cells in S and G_2_/M cell cycle phases (lower right) and the total percentage of BrdU-positive cells (upper right) are shown in each dot plot. Representative results from one of 3 independent experiments.

Our lab previously developed a primary T cell model of acute HIV-1 infection in quiescent CD4 T lymphocytes [21]. Our first aim in this study was to determine whether we could adapt and modify this model to investigate the establishment of HIV latency. To characterize the cellular effects of induced T cell activation following HIV infection of resting cells, viable cell counts were performed on CD4 T cell aliquots immediately after infection (day 0) and sequentially on days 4, 7, 10, and 14 following stimulation by T-cell receptor (TCR) cross-linking. Fig 1A outlines the design of these experiments, showing the timing of HIV infection and the sequential culture manipulations performed. For comparison, autologous uninfected CD4 T cells were cultured and monitored in parallel in each experiment. The uninfected CD4 T cell cultures went through approximately 7 to 10 days of a proliferative expansion phase, reaching maximum cell numbers by day 10 (Fig 1B). In contrast, the infected cell cultures showed no increase in overall relative cell numbers, but rather a decrease during the same time period (Fig 1B). This was likely the result of cell death and turnover due to HIV cytopathic effects during productive viral replication. Following the cell proliferation phase, both uninfected and infected cell cultures went through a contraction phase of activation-induced cell death during days 10 to 14 (Fig 1B), when exogenous cytokines were no longer being replenished (Fig 1A). Such activation-induced cell death has been ascribed to an apoptotic mechanism [32] and the added variable of HIV infection seemed to have substantially increased these effects. Flow cytometric assessment of cellular DNA content at the end of culture showed a much larger portion of cells containing fragmented DNA in the infected cultures (Fig 1C).

To confirm that the CD4 T cells derived from our *in vitro* model were in a quiescent resting state at the end of culture, and could therefore be used to model latent infection, cells were harvested at day 14 and examined for their activation status by cell cycle analysis. Cell aliquots from the parallel infected and uninfected cultures were exposed overnight to the thymidine analog bromodeoxyuridine (BrdU), then fixed and stained for BrdU incorporation in combination with 7-AAD staining for DNA content. Flow cytometry analysis demonstrated that the vast majority of cells (>98%) had returned to a resting G_0/1_ state by the end of culture (Fig 1C). Although a small percentage of cells appeared to be in the S and G_2_/M stages of cell cycle in both the infected and control cultures, little to no BrdU incorporation was evident (Fig 1C). In three repeated experiments with infected cells from different donors, the percentage of cells in S plus G_2_/M phases ranged from 0.8 to 2.8% and total BrdU incorporation, from 0.4 to 1.1%. These minor subsets were evident from initiation of culture, prior to cell stimulation. Together, the data suggested that such cell subpopulations were not actively synthesizing DNA and may have been suspended in a G_2_ or S cell cycle phase for an indeterminate amount of time. We concluded that the overall state of these CD4 T cells, after 14 days of culture, represented functional quiescence, as reflected by their phenotypic similarity to the freshly isolated primary cells at initiation of culture.

### Kinetics of HIV replication induced during *in vitro* cell culture

**Fig 2:**
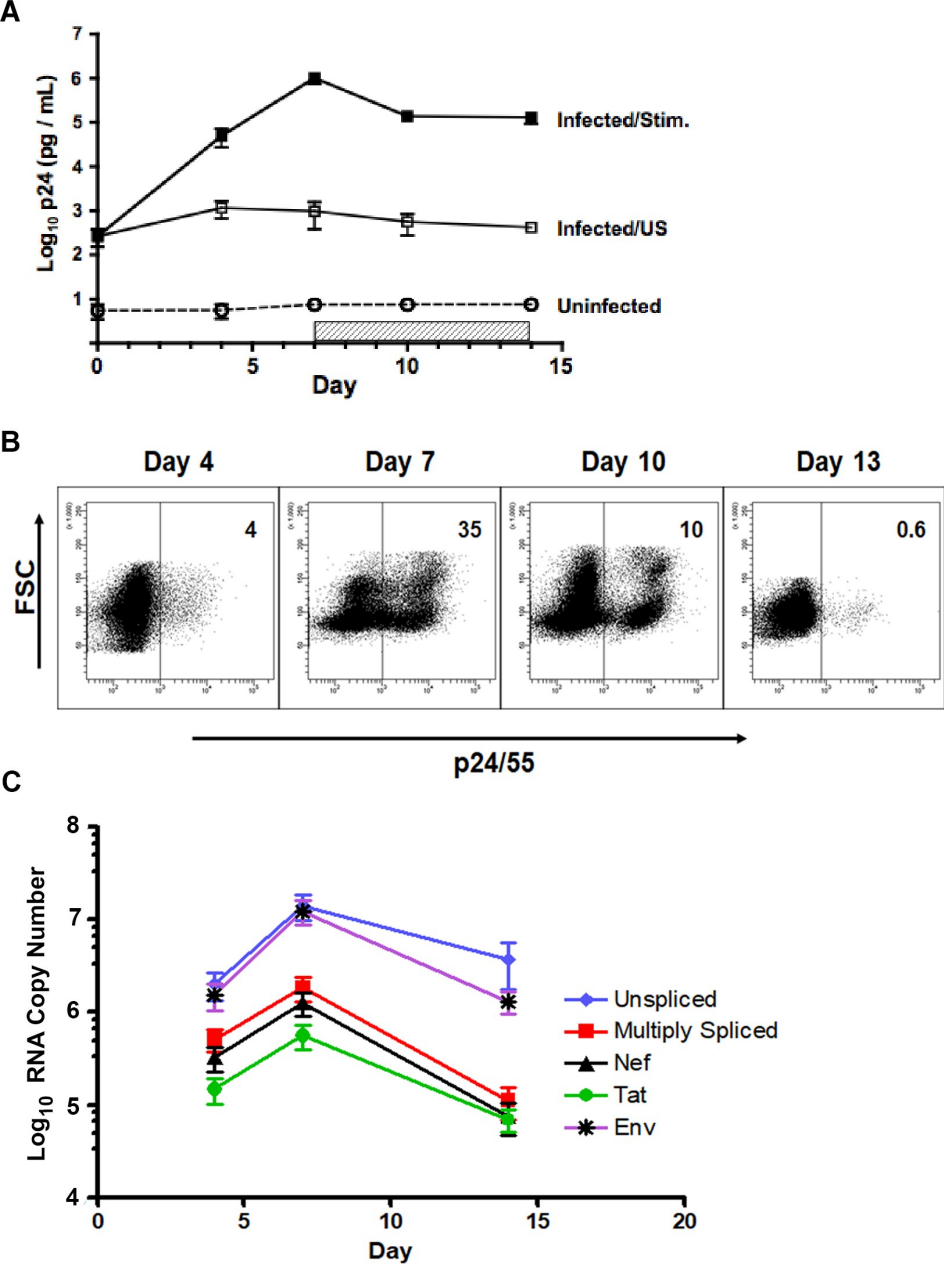
Kinetics of HIV replication induced during *in vitro* cell culture. (A) Productive HIV replication was analyzed in parallel with the induction of cell proliferation (Fig 1B). The NL4-3 infected CD4 T cells (Infected/Stim) and uninfected control cells (Uninfected) were stimulated and maintained in culture for 14 days, according to the experimental design shown in Fig 1A. The horizontal hatched bar indicates the addition of antiretroviral Indinavir (HIV protease inhibitor) to culture (days 7–14). A separate portion of infected cells was maintained in culture medium alone, without the addition of stimulating agents, cytokines or Indinavir (Infected/US). Aliquots of culture supernatant were removed on days 4, 7, 10, 14; and assayed for levels of secreted p24 by ELISA. Results shown: cumulative mean ± SEM for 5 independent experiments (cells from same donors used for Fig 1B). (B) Representative experiment showing serial analysis of intracellular expression of HIV Gag (p24/55) in a culture of infected, TCR-stimulated CD4 T cells; Indinavir added on day 7. Results depicted by flow cytometry dot plots of forward scatter, FSC (cell size) versus intracellular Gag (ICp24/55) staining. The positive cursor was set according to the fluorescence threshold of the uninfected cell control, stained in parallel with anti-p24/55 antibody. The percentage of cells, expressing intracellular Gag (ICp24/55) at each time point is given in the upper right corner of each plot. Representative results from one of 5 independent experiment, using cells from different donors. (C) Infected and stimulated CD4 T cells were cultured, with Indinavir present from day 7 through day 14. Cell aliquots were taken on days 4, 7 and 14 for RT-qPCR analysis of five different species of HIV transcripts. RNA copy numbers were normalized to 25 ng total RNA input. Results show the mean ± SEM of 3 experiments, using cells from different donors.

Productive virus replication was tracked by measuring the secretion of soluble p24 into culture supernates, using standard ELISA analysis. The levels of HIV production increased rapidly over 7 days, reaching peaks of approximately a thousand fold over initial baseline (Fig 2A). To determine the frequency of infected cells, intracellular Gag (ICp24/55) expression was followed sequentially by flow cytometry analysis. In a representative experiment shown in Fig 2B, cells expressing ICp24/55 were easily detected by day 4 of culture (4%) and increased to approximately one third of the culture (35%) at the peak of infection, on day 7. During the contraction phase of cell proliferation and in the presence of antiretroviral treatment, the proportion of ICp24/55-positive cells decreased (10%) until only a very small percentage could be detected (0.6%) at the end of culture. In a series of five repeated experiments, peak CD4 T cell infection on day 7 averaged 26 ± 4% (mean ± SEM). Flow cytometry analysis also demonstrated that ICp24/55 expression increased initially in the larger blasting cell fraction (Fig 2B, FSC), but gradually shifted to the subpopulation of small cells, which accumulated over time. By both secreted (p24) and intracellular (p24/55) measures of expression, HIV infection peaked at 7 days following induction of cell stimulation, coinciding with the approximate peak of cell proliferation. The HIV protease inhibitor, Indinavir was added on day 7 to block further spread of infection in culture and reduce potential super-infection events. At the end of culture on day 14, when the majority of cells had returned to a resting state, productive HIV replication had ceased.

Because findings from *in vivo* studies of HIV latency have described the variable presence of viral transcripts in recovered quiescent CD4 T cells [33], we characterized cells taken sequentially from our *in vitro* model for detectable levels of viral RNA transcription. For this purpose, a quantitative reverse transcriptase-PCR (RT-qPCR) method was developed (refer to Materials and Methods for details) that was based on the general technical approach described previously by Fischer et al. [34]. The assay quantified five different RNA species produced during NL4-3 infection: unspliced/gag, singly-spliced/env, multiply-spliced, nef-specific, and tat-specific. Copy numbers of each viral RNA species increased over time following productive infection, reaching peak levels at day 7 and then, decreasing out to day 14 (Fig 2C). As expected, the kinetics of viral RNA transcription paralleled those observed for intracellular Gag (ICp24/55) expression. The relative hierarchy of transcript frequency remained constant throughout the sampling time, with unspliced and singly-spliced env transcripts being the most abundant, followed by multiply-spliced, nef, and tat species (Fig 2C). Even though the vast majority of cultured cells had returned to a resting state by day 14, all viral transcripts remained detectable at that time, with the unspliced and singly-spliced species appearing the most stable.

### Establishment of latent infection

To determine the number of latently infected cells that were established in our culture model (Fig 1A), we quantified both the number of integrated HIV DNA copies and the number of cell-associated replication-competent infectious units (refer to Materials and Methods for complete technical details) in 6 replicate experiments, using cells isolated from different donors. At cell harvest on day 14, the average number of proviral copies detected per 500 ng of isolated genomic DNA (equivalent of 80,000 primary T cells) was approximately 18,000 copies (Table 1). However, the majority of these detected integrants were not induced into productive virus replication by a secondary round of TCR stimulation, as demonstrated by the number of replication-competent infectious units per million cells (IUPM) detected: approximately 4,000 based on limiting dilution co-culture analysis (Table 1). The calculated mean ratio of HIV provirus per infectious unit (IU) was 103 (Table 1), which falls within the lower range previously reported for latently infected T cells isolated from persons with HIV (100–1,000) [35].

**Table 1:** Quantification of HIV latent infection at the end of culture.

	Integrated HIV (copies/500 ng)^b^	IUPM^c^	Integrants per IU^d^
Mean^a^	18,405	4,121	103
Range	2,738–48,420	162–11,212	12–211

^a^CD4 T cells were infected with NL4-3 (MOI = 0.01 TCID_50_) on Day 0; Indinavir was added on Day 7 and maintained throughout the remainder of culture. On Day 14, cell aliquots were taken for: i) isolation of genomic DNA and qPCR determination of HIV integrant copies, and ii) restimulation and quantification of cell-associated infectious units (IUPM) by limiting dilution viral outgrowth assay (QVOA). Results are derived from 6 independent experiments, using cells from different donors.

^b^500 ng genomic DNA = 80,000 cell equivalents

^c^IUPM, infectious units per million cells

^d^IU, infectious units. Integrants per IU were obtained for each experiment (Range) by calculating the integrated HIV copies per cell (e.g. copies per 500 ng DNA divided by 80,000 cell equivalents) followed by division with the sample associated IUPM. The average value (Mean) was then derived from these individual ratios.

### Infection prior to T cell stimulation results in the greatest number of latently infected cells

**Fig 3:**
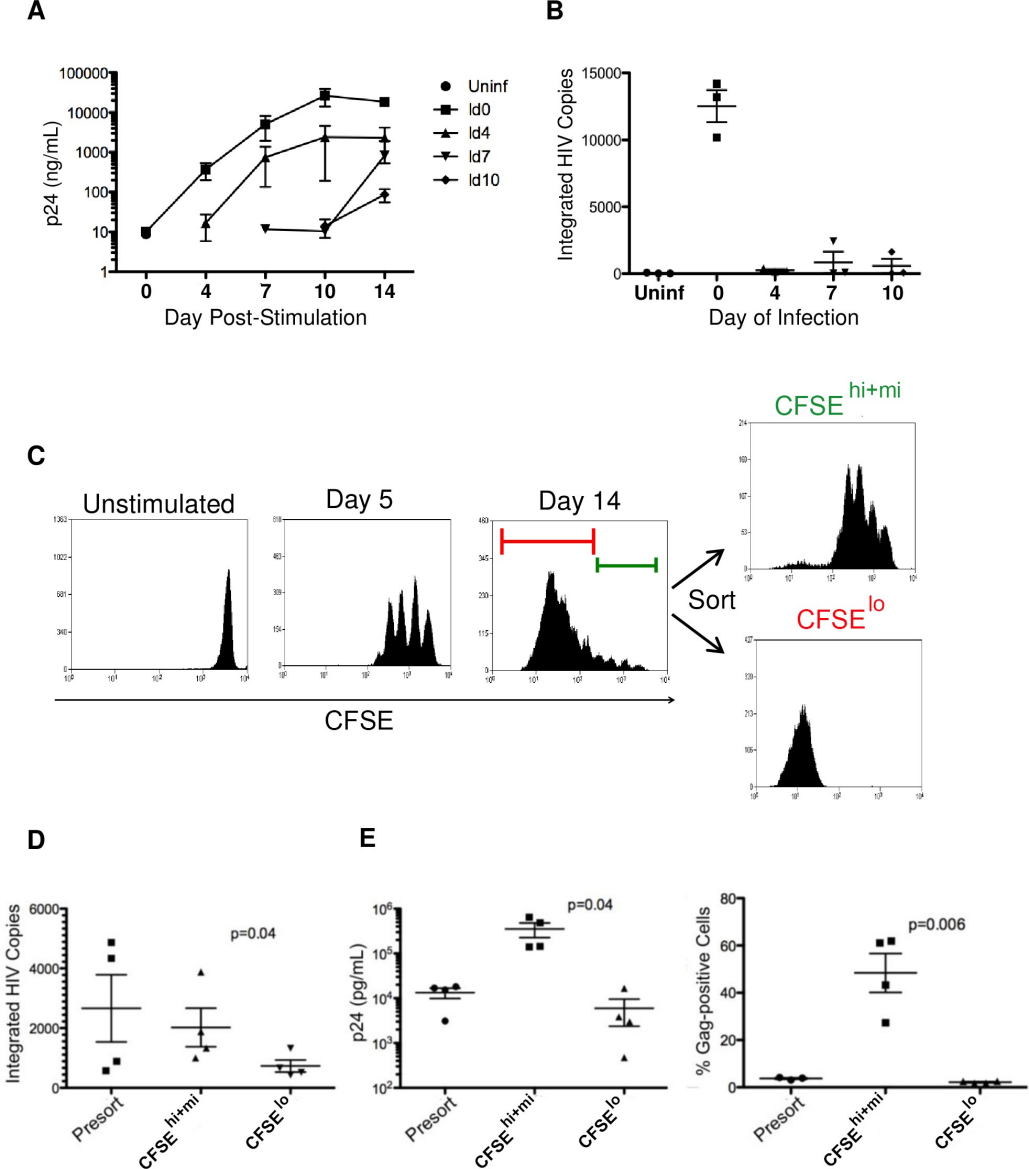
Cell stimulation status at the time of infection and the amount of cell proliferation following infection impact the establishment of HIV latency. Aliquots of freshly isolated primary CD4 T cells were infected with HIV at different time points relative to the addition of activated stimulus: before (day 0) or following (days 4, 7, 10) initial TCR stimulation. Antiretroviral drugs were not added to culture in these experiments. (A) Productive virus replication, following infection at varying time points after cell stimulation (day 4) was monitored sequentially over time in culture (days 0 to 14) for the levels of secreted p24 production by ELISA analysis. (B) Cell aliquots were taken at the end of culture (day 14), to extract genomic DNA and perform qPCR analysis for the quantification of integrated HIV DNA copies. Sample legend (A and B) for time of HIV infection relative to the addition of activation stimulus: uninfected control, Uninf; infected on day 0, Id0; on day 4, Id4; on day 7, Id7; on day 10, Id10. Data shown (A and B) represent the mean ± SEM from 3 independent experiments, using cells from different donors. In a separate set of cell-sorting experiments (C, D and E), our standard 14-day cell model of HIV latency (Fig 1A) was used to examine the influence of infected cell proliferation on the capacity to establish latent infection. Prior to cell infection and stimulation, the isolated CD4 T lymphocytes were stained with CFSE dye to track progressive cell divisions during culture. (C) Diagram of experimental design for the identification and sorting of cell subsets with different proliferation profiles (representative graphics taken from one of 4 replicate experiments). Nevirapine (RT inhibitor) was added on day 5. At the end of culture (day 14), cells were sorted, based on their CFSE content and proliferation profile (C), into subsets that: i) had divided only a few times (CFSE ^**hi+mi**^ / far-right top panel), using a sorting gate depicted by the green bar in the Day 14 histogram (right panel), which was determined by the cell division profile seen on Day 5 (middle panel histogram); or ii) had divided many times following day 5 (CFSE ^**lo**^ / far-right bottom panel), using the red bar sorting gate depicted in Day 14 histogram. (D) Aliquots of infected cell subsets, recovered at day 14 from 4 replicate sorting experiments were analyzed for copies of integrated HIV DNA per 500 ng total genomic DNA (80,000 cell equivalents). (E) Additional aliquots from the recovered infected cell subsets were restimulated using plate-bound anti-CD3 + anti-CD28. Induction of soluble p24 release (left graph) and percent intracellular Gag (ICp24/55) expression (right graph) were quantified at 7 days following the secondary reactivation by TCR stimulus. Individual data points, with mean and SEM, are shown for 4 experiments using cells from different donors.

After a thorough characterization of our *in vitro* cell culture model and its ability to generate latently infected cells, we proceeded to employ it to determine the phase of T cell activation at which HIV infection is most likely to progress to latency. Aliquots of 10 x 10^6^ CD4 T cells were infected at different time points relative to the addition of cell-activating stimulus: approximately 18–20 hrs before (day 0) and 4, 7, and 10 days following TCR stimulation with immobilized anti-CD3 + anti-CD28 antibodies. After HIV infection at the selected time points, each cell aliquot was cultured separately until harvest at 14 days following the initial addition of stimulus. Active virus replication, measured by soluble p24 production, was greatest in cells that were infected immediately (day 0) before stimulation (Fig 3A). These data confirmed original observations from our early reported studies [21, 36]. Cells that were exposed to infectious HIV before the addition of TCR activation stimulus produced approximately ten fold more virus at the peak of replication than did cells, infected 4 days following addition of stimulus. When infection was initiated at 7 or 10 days following cell stimulation, minimal productive HIV replication was detected (Fig 3A). After 14 days in culture when cells had returned to a resting state, cellular genomic DNA was extracted and analyzed by qPCR for numbers of integrated copies of HIV DNA, as outlined in Table 1. In parallel to the results obtained for productive virus replication, cells infected before stimulation (day 0) also contained the greatest numbers of integrated HIV DNA copies at the end of culture (Fig 3B). Interestingly, exposure to infectious HIV during the height of cell proliferation (4–7 days post-stimulation) resulted in the least amount of integrated viral DNA, which was retained in CD4 T cells that survived to day 14.

### Latent infection is established preferentially in cells that proliferate the least following TCR stimulation

Latently infected cells, collected at the end of culture in our *in vitro* model, could have derived from T cells that survived initial infection and multiple rounds of cell division or resulted from later rounds of infection, as virus spread through the proliferating cell culture. To address this question, cell proliferation of the infected culture was tracked using the viable membrane dye carboxy-fluorescein diacetate succinimidyl ester (CFSE) and serial flow cytometry analysis to monitor progressive cell divisions and shifting CFSE dye expression patterns over time. Five days after initiation of cell stimulation, the reverse transcriptase (RT) inhibitor Nevirapine was added to the culture to prevent subsequent virus spread and new rounds of infection. At 14 days following cell infection and stimulation, CD4 T cell subpopulations that had undergone varying degrees of proliferation, were isolated by flow cytometric cell sorting, based on their CFSE staining profiles (Fig 3C). An aliquot of the cultured cells, taken prior to sorting and the two main recovered cell subsets (CFSE^**hi+mi**^ and CFSE^**lo**^) were analyzed for amounts of integrated HIV DNA (Fig 3D), and the ability to replicate virus, upon a second round of cell stimulation (Fig 3E).

Surprisingly, the subpopulation containing cells that did not divide or divided only a few times (CFSE^**hi+mi**^) during culture, contained higher levels of integrated HIV DNA copies (Fig 3D) than did the portion of cells which had divided many times (CFSE^**lo**^). The presence of inducible HIV in each of these two recovered subpopulations (Fig 3C) was evaluated by induced production of soluble p24 and intracellular Gag (ICp24/55) expression at 7 days following restimulation with immobilized anti-CD3 + anti-CD28 antibodies (Fig 3E). Under conditions of spreading HIV infection during the second round of stimulation, cells that had proliferated the least during initial culture (CFSE^**hi+mi**^) secreted 1–3 more p24 (Log_10_) and generated 10–30 fold higher frequencies of infected cells expressing intracellular Gag (ICp24/55) than did cells, which had originally divided many times (CFSE^**lo**^) (Fig 3E).

### Within a heterogeneous cell proliferative response, latent infection can be established in cells that do not divide in response to TCR stimulation

**Fig 4:**
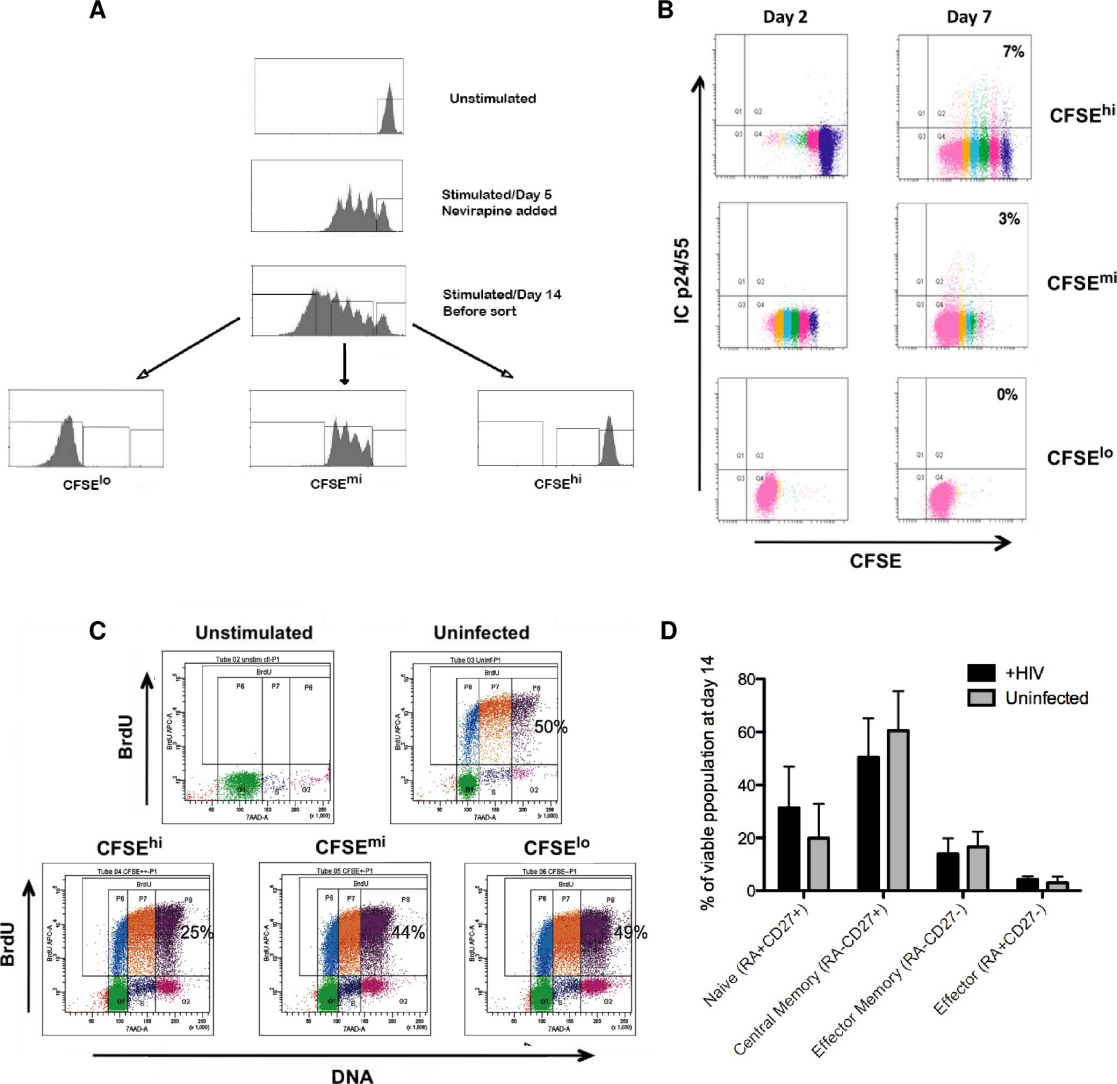
Latent infection is established in CD4 T cells that do not divide in response to TCR stimulation. (A) Representative example of the experimental design for identification and sorting of cell subsets with different proliferation profiles. CD4 T lymphocytes were prepared and cultured as described in Fig 3C. Nevirapine, RT inhibitor was added on day 5. At the end of culture (day 14), cells were sorted based on their CFSE content and proliferation profile into subsets that had: i) not divided (CFSE^**hi**^); ii) divided only a few times after day 5 (CFSE^**mi**^); or iii) divided many times after day 5 (CFSE^**lo**^). (B) Infected cell subsets, recovered from sorting on day 14, were washed and restimulated with immobilized anti-CD3 + anti-CD28 to induce a second round of productive virus replication. Each recovered cell subset was analyzed by flow cytometry for expression of intracellular Gag (ICp24/55) at 2 and 7 days, following reactivation. Representative data, from one of 4 experiments, are shown. (C) Cell subsets isolated at the end of the initial culture phase (day 14) were tested for their capacity to respond to a second TCR stimulation. On day 4 following restimulation, aliquots from the uninfected control culture and each infected cell subset were analyzed by flow cytometry for cell status (7-AAD staining of DNA content) and level of active DNA synthesis (2 hr pulse with BrdU). Representative data, from one of 3 experiments, are shown. (D) Bar graph of infected (black) and uninfected (gray) cell subpopulations that were stained on day 14 for CD45RA and CD27 co-expression. Bars show the proportions of different T cell maturation phenotypes found in the CFSE^**hi**^ subset of cultured cells that had not proliferated. Results depict the mean + SD of 4 experiments, using cells from different donors.

Next, we sought to determine whether latent infection was established in the minor cell subset that had not undergone any detectable proliferation during culture. A second set of cell sorting experiments, based on CFSE staining profiles was performed that focused on cells exhibiting no cell division (Fig 4A), as demonstrated by cells not showing any dilution of the brightest CFSE profile (4A unstimulated / top panel). With this experimental design, three cell subsets were recovered: CFSE^**hi**^, CFSE^**mi**^, and CFSE^**lo**^ (Fig 4A). The infected cell subset without detectable cell division (CFSE^**hi**^) had the greatest capacity to generate functional virus following a second round of TCR stimulation, as shown by the greater percentages of cells that expressed intracellular Gag (ICp24/55) at 7 days following reactivation (Fig 4B). In addition, the cell subset lacking evident proliferation (CFSE^**hi**^) contained integrated HIV (Table 2) at levels comparable to that of cells which had divided only 4–5 times during culture (CFSE^**mi**^). The decreased ability to induce virus replication from the cell subset that had initially responded with vigorous proliferation (CFSE^**lo**^) was not due to any detectable deficiency in proliferative capacity among the recovered cell subpopulations. All recovered cell subsets responded well during a second round of induced cell stimulation, as shown by levels of BrdU incorporation (Fig 4C). The only noted variation was with the recovered CFSE^**hi**^ subpopulation that exhibited a somewhat lower relative response to the second round of TCR stimulation (Fig 4C, left bottom panel).

**Table 2:** Integrated HIV in sorted CFSE-stained cell subsets.

	Integrated HIV copies/500ng DNA^a^			
	Presort^b^	CFSE^hi^	CFSE^mi^	CFSE^lo^
Donor 1	568	387	438	223
Donor 2	24,266	2,632	3,672	1,179
Donor 3	1,267	732	1,378	319
Mean	8,701	1,251	1,829	574

Results derived from experiments that used design outlined in Fig 4A. Sorted cell subsets recovered from end of culture on day 14. Data obtained from three independent experiments, using cells from different donors.

^a^500 ng genomic DNA = 80,000 cell equivalents

^b^Presort aliquots taken from infected bulk culture just prior to FACS

To examine whether exposure to HIV infection in the non-proliferating cell subset (CFSE^**hi**^) altered the relative proportions of T cell major maturation phenotypes in the cultures recovered at day 14, additional flow cytometry analysis was done. Co-expression of CD45RA and CD27 cell membrane differentiation antigens was used to identify the four major maturation subset categories: naïve (45RA^**+**^27^**+**^), central memory (45RA^**-**^27^**+**^), effector memory (45RA^**-**^27^**-**^), effector (45RA^**+**^27^**-**^). As shown in Fig 4D, all four major phenotype subsets were present, and no significant differences in their proportions were found between the HIV infected and uninfected CFSE^**hi**^ cultures (p-values for uninfected and infected group comparisons ranged from 0.06 to 0.19, using Wilcoxon signed rank test).

### During induced response to TCR stimulus, the non-dividing CD4 T cell subset appears to be sub-optimally activated

**Fig 5:**
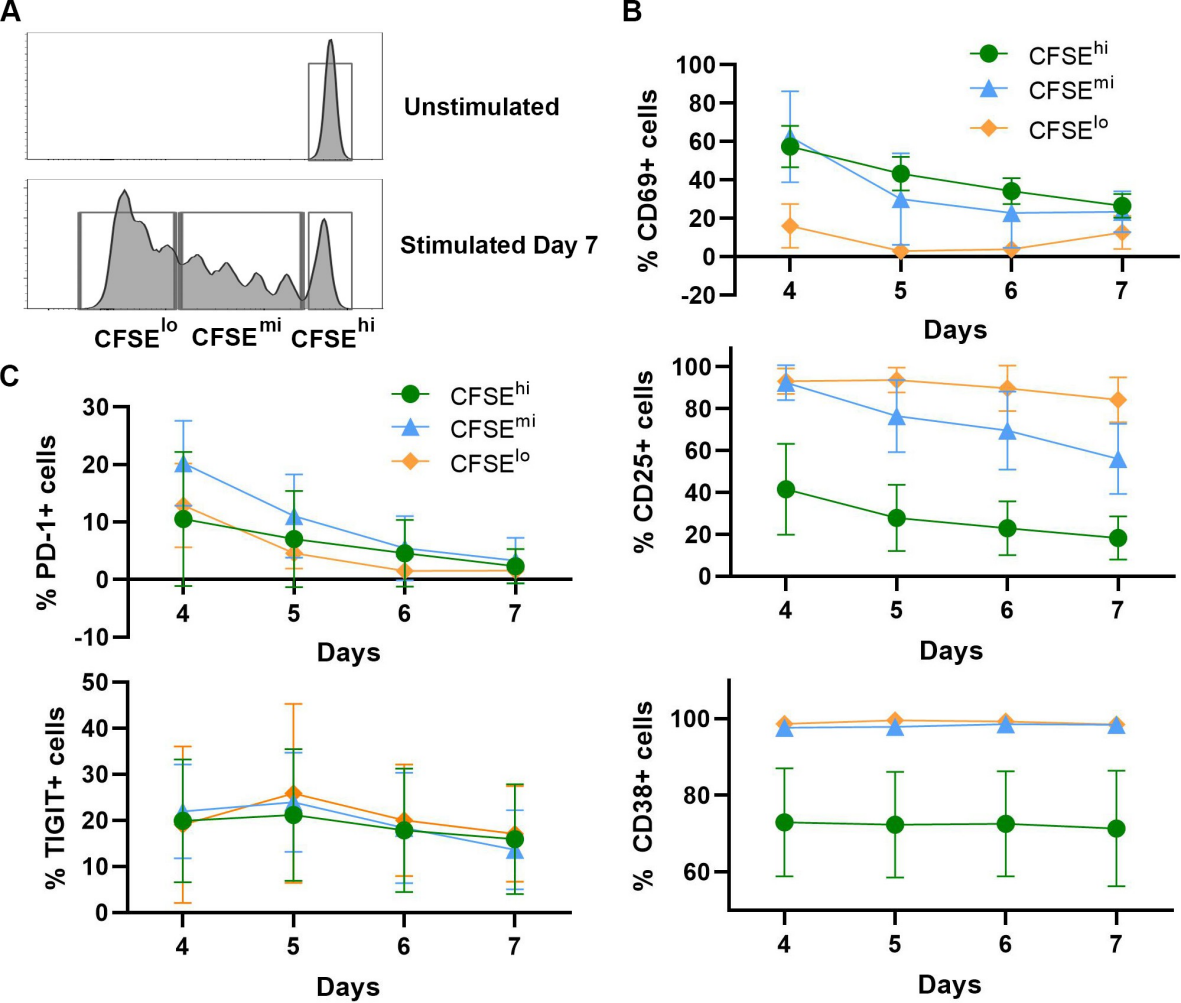
Characterization of CD4 T cell subset responses following TCR stimulation. Aliquots of isolated CD4 T cells were stained with CFSE dye, infected with HIV, and activated with immobilized anti-CD3 + anti-CD28 antibodies, as described in Fig 4A, except no antiretrovirals were added. After 4 days, the cells were removed from TCR stimulus, washed, resuspended in fresh medium supplemented with IL-2 and IL-15, and transferred to new culture plates. Cell samples were collected daily on days 4 to 7, for flow cytometry expression analysis of antigen markers associated with activation (CD69, CD25 and CD38) and exhaustion (PD-1, TIGIT). (A) The gating strategy based on number of cell divisions. The far-right cell population peak (bottom panel) was set as the non-dividing subpopulation (CFSE^**hi**^), based on the unstimulated cell profile (top panel). Cells that divided 1–4 times composed the CFSE^**mi**^ subset, and cells that went through more than 4 divisions composed the CFSE^**lo**^ subset (bottom panel). Percentages of cells expressing each activation (B) and exhaustion (C) associated marker were determined for each of the three defined subpopulations (CFSE^**hi**^, CFSE^**mi**^, and CFSE^**lo**^) throughout the 7-day time course. Results depict the mean values ± SD (error bars) of 3 experiments, using cells from different donors.

To further characterize the phenotypes of cell subpopulations that were recovered after induced stimulation, expression levels of T cell markers, associated with activation (CD69, CD25 and CD38) and exhaustion (PD-1, TIGIT), were evaluated in the non-dividing (CFSE^**hi**^) and dividing (CFSE^**mi**^ and CFSE^**lo**^) cell subsets over time following TCR stimulation (days 4–7). The gating strategy used for flow cytometry analysis in these experiments is illustrated in Fig 5A. Among the markers tested, the early activation marker, CD69 was expressed on a greater percentage of non-dividing cells (CFSE^**hi**^) and cells with very limited divisions (CFSE^**mi**^), in comparison to cells that divided many times (CFSE^**lo**^). Over time, the magnitude of these differences decreased until the end of culture at day 7 (Fig 5B, top panel). In contrast, markers of later phase cell activation, CD25 (IL-2Rα) and CD38 (thymic-associated maturation) were expressed on a majority of cells (90–95%) that underwent division (CFSE^**mi**^ and CFSE^**lo**^) but were detected on a substantially smaller percentage (40–70%) of the non-dividing (CFSE^**hi**^) cell subset (Fig 5B, middle and bottom panels). These large differences in CD38 expression between cell subpopulations remained fairly constant over time (Fig 5B, bottom panel), while the proportion of cells expressing CD25 decreased gradually (loss of ~20%), especially in the CFSE^**mi**^ and CFSE^**hi**^ subsets (Fig 5B, middle panel). For antigen markers associated with cell exhaustion, PD-1 expression was detected at day 4 on a small percentage of cells in all three subsets, and the subpopulation with a very limited proliferation profile (CFSE^**mi**^) showing the greatest expression (Fig 5C, top panel). Over time, PD-1 expression decreased gradually until any differences between subsets disappeared by day 7. The TIGIT marker, associated previously with latent HIV infection [37, 38], showed a similar expression pattern in all three cell subsets and remained stable over time (Fig 5C, bottom panel).

Taken together, these results are consistent with the idea that the small subpopulation of non-dividing cells, found in our model of HIV latency were below a stochastic threshold [39] for induction of cell proliferation by either the format of TCR cross-linking or the levels of exogenous cytokine (IL-2, IL-15) additions, which were used in our particular culture system and therefore, represents a “sub-optimally activated” cell subset following the initial stimulation event. However, we also performed other experiments that demonstrated there is no inherent deficiency in proliferative capacity in this non-dividing cell subset (CFSE^**hi**^). When these cells, recovered at day 14 were exposed to the second round of TCR stimulation, they produced a vigorous response (Fig 4C). On the other hand, the lack of demonstrable major increases in the expression of antigen markers associated with cell exhaustion in this group of experiments argues against that variable as an important contributor here or may be attributable to the short observation time used (4–7 days following TCR stimulation) in this experimental design.

### In this CD4 T cell model, HIV Vpr protein does not contribute to establishment of viral latency

**Fig 6:**
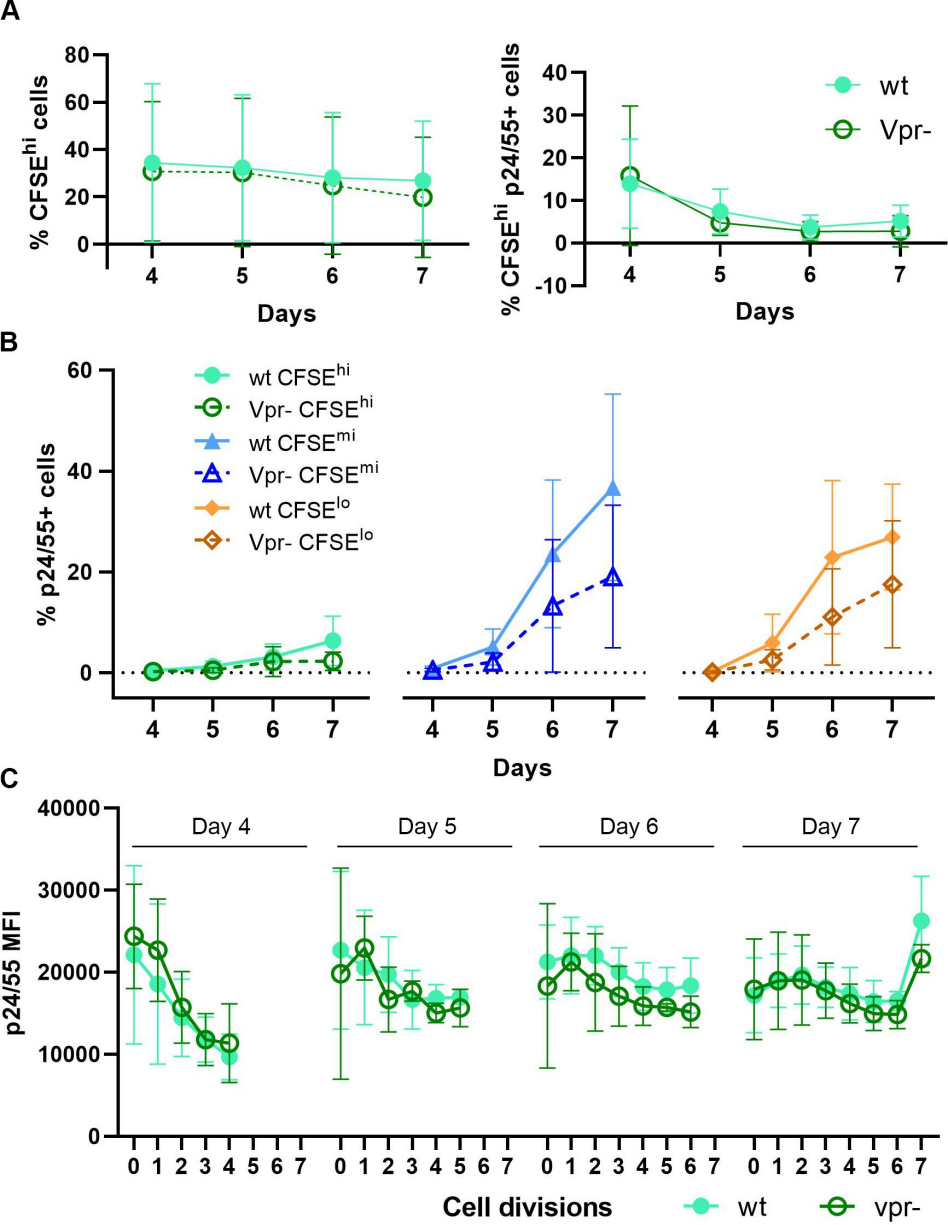
Potential influence of Vpr in the establishment of latent HIV infection in primary CD4 T cell cultures, following TCR stimulation. Experimental design, based on that described in Fig 5. Aliquots of isolated CD4 T cells were stained with CFSE dye, infected with isogenic HIV clones, and activated with immobilized anti-CD3 + anti-CD28 antibodies. Cell samples were infected in parallel (MOI, 0.01 IU) with wild type (wt) NL4-3 and its vpr-mutated clone (vpr**-**). After 4 days of activation, the cells were removed from TCR stimulus, washed, resuspended in fresh medium supplemented with IL-2 and IL-15, and transferred to new culture plates. No antiretroviral drugs were added. Cell samples were collected daily on days 4 to 7 to evaluate HIV replication, using flow cytometric analyses with the gating strategy and identification of CFSE-stained cell subset proliferation profiles, as depicted in Fig 5A. (A) Proportions of non-dividing cells (CFSE^**hi**^) in the infected cell population that expressed Gag (ICp24/55) were compared between the cultures infected with wild type (wt) and vpr-mutated clone (vpr**-**) of NL4-3, throughout the 7-day time course. (B) Percentages of cells expressing intracellular Gag were determined for each of the three defined cell subpopulations (CFSE^**hi**^, CFSE^**mi**^, CFSE^**lo**^), comparing the wt and vpr**-** infected cultures over time and increasing cell divisions. (C) In the non-dividing cell subpopulation (CFSE^**hi**^) that was present throughout culture (6B), the relative amount of Gag protein expressed per cell (mean fluorescence intensity, MFI) was measured initially and followed through subsequent cell divisions in samples taken on days 4 to 7, comparing the wt and vpr**-** infected cultures. Results depict the mean values ± SD (error bars) of 3 experiments, using cells from different donors.

One possible explanation for the high frequency of latent infection that was detected in the non-dividing cell subpopulation (CFSE^**hi**^) is the potential influence of the Vpr accessory protein of HIV, which has the associated functional property to induce cell cycle arrest [40]. To examine this possibility, we performed an additional small set of experiments (Fig 6) that compared infection using wild type NL4-3 with that using an isogenic viral clone, containing mutated non-functional vpr [40, 41] (refer to Materials and Methods for virus details). If Vpr expression produced a detectable influence on the establishment of viral latency in our cell model, we would expect that a greater proportion of infected cells should accumulate within the non-dividing cell subset over time in cultures infected with wild type (wt) NL4-3, compared to cultures infected with the vpr mutant (vpr**-**). However, the results from these experiments showed that both the proportion of non-dividing cells and the proportion of cells, expressing intracellular Gag protein (ICp24/55) in the non-dividing cell subpopulation (CFSE^**hi**^) remained constant throughout the monitored time course (Fig 6A). Consistent with previously published findings [42], proportions of Gag expressing cells were slightly greater when cells were infected with wild type, compared to vpr-mutated HIV; the reduction in infectivity, however was independent of extent of cell proliferation (Fig 6B). Furthermore, the presence of wild type vpr did not alter the expression of T cell phenotype markers associated with either activation (S1A Fig) or exhaustion (S1B Fig); nor did it affect the loss of CD4 receptor from the cell plasma membrane (S1C Fig) during active viral replication, as is commonly reported. Together, these results suggest that enrichment for latent infection in the non-dividing cell subpopulation cannot be attributed to the inhibitory effect of Vpr on cell cycle progression, but rather is more likely due to increased overall expression of HIV accessory proteins during productive wild type virus infection, especially those linked to apoptotic induced cell death (i.e. Vpr, Tat, Nef) [43].

The increases in viral Gag production (ICp24/55) seen in our *in vitro* system (Fig 6B) can serve as a reference point for the expected patterns of increasing overall viral protein production. In addition to tracking the proportion of cells that expressed Gag in the parallel infected cultures (wt and vpr**-**), the relative amount of intracellular Gag protein per cell (mean fluorescence intensity, MFI) was monitored also. To address our central concern regarding the influence of Vpr on kinetics of HIV replication, attention was focused on the subset of non-dividing cells (CFSE^**hi**^), which was present in each cell aliquot taken from cultures on days 4 to 7, and followed through subsequent cell divisions (Fig 6C). The amount of initial Gag expression (MFI; 0 cell divisions), present in the CFSE^**hi**^ subset taken from culture at day 4 was relatively high, but decreased rapidly during several cell divisions (Fig 6C, far-left panel). In contrast, high Gag expression detected initially (MFI; 0 cell divisions) in the CFSE^**hi**^ subset, taken at day 7 remained relatively constant over many subsequent cell divisions (Fig 6C / far-right panel). Results from this set of experiments, which demonstrated infected T cell cultures contained both greater proportions of Gag-positive cells (Fig 6B) and higher levels of Gag expression per individual cell (Fig 6C) following TCR stimulation, support the premise that increased T cell division preferentially drives the death of productively infected cells and reduces establishment of latent infection. Importantly, Gag MFI profiles over time in the cultures, infected with vpr-mutant tracked almost perfectly with those of wt virus-infected cultures (Fig 6C); therefore, indicating the influence of Vpr in the establishment of viral latency in our CD4 T cell model is highly questionable.

In summary, all these combined results demonstrated that among CD4 T cells, derived from our *in vitro* culture system, HIV latent infection was generated at a higher frequency in those cells that become only minimally activated while in the presence of fully activated and productively infected cells. Therefore, we hypothesized that a minimal to moderate level of activation may be sufficient to drive HIV integration into host cell DNA without being strong enough to induce productive, cytolytic virus replication. Such minimally activated CD4 T cells would then be expected to preferentially survive with persistent or latent infection.

### Latent infection is established directly in non-dividing CD4 T cells that are “bystanders” to productive HIV infection

**Fig 7:**
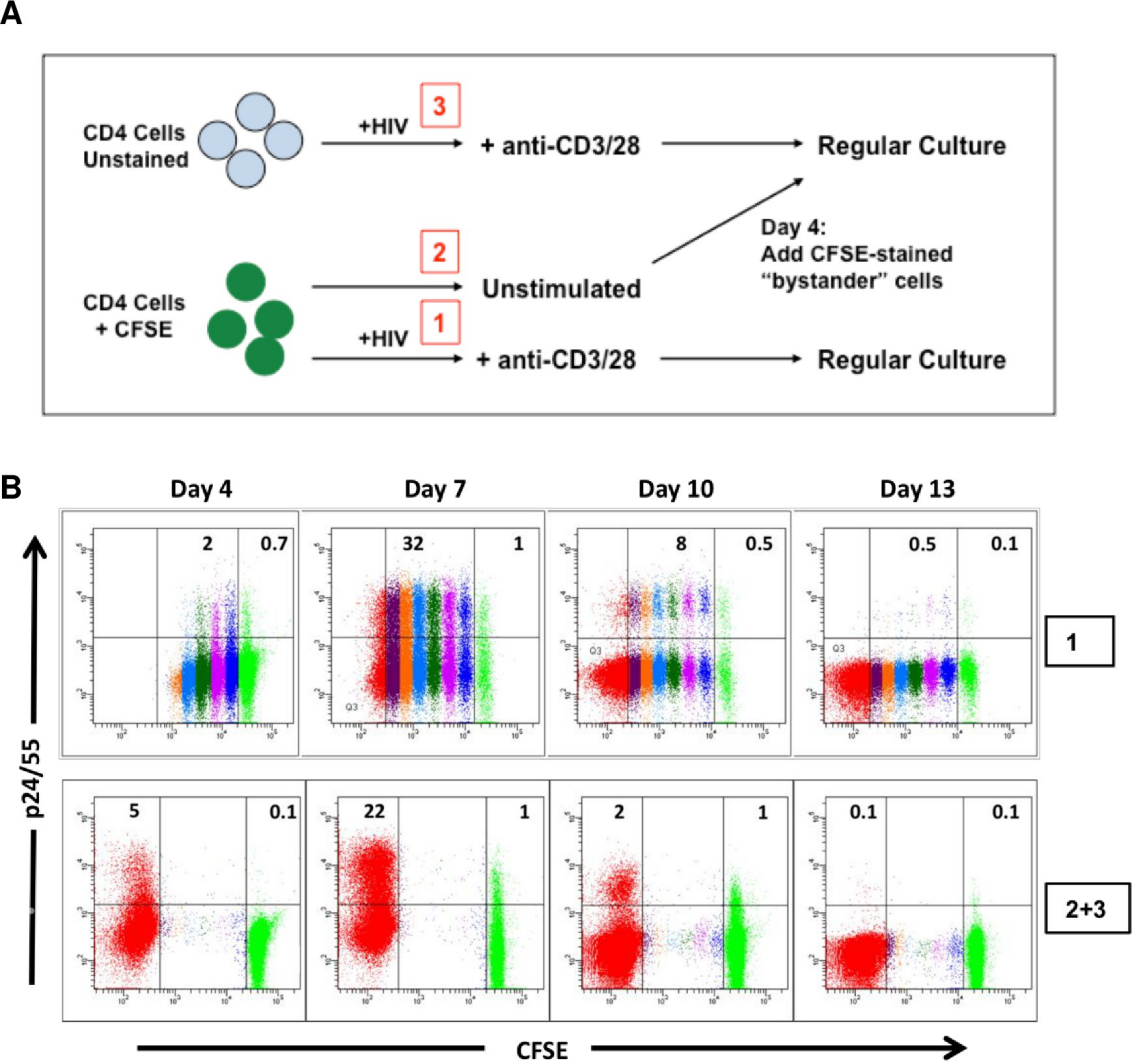
Direct establishment of latent HIV infection in non-dividing “bystander” CD4 T cells during co-culture with productively infected cells. (A) Schematic diagram of experimental design. After isolation of CD4 T lymphocytes, a portion of the cells was stained with CFSE dye, and the remaining cells were left unstained. The unstained cells were infected, stimulated, and cultured according to the standard *in vitro* model protocol (condition #3 in diagram). Following 4 days in culture, the unstimulated, uninfected, CFSE-stained cells (condition #2 in diagram) were mixed with the infected, stimulated cells in a 1:1 ratio. The cell mixture was cultured for an additional 10 days in the presence of cytokines; Indinavir was added at day 7. For comparison, a separate portion of the CFSE-stained cells was infected, stimulated, and cultured alone in parallel (condition #1 in diagram). (B) The cells were monitored for intracellular Gag (p24/55) expression and degree of cell division (CFSE content) until the end of culture. Top row: CFSE-stained, infected, and stimulated cells. Bottom row: CFSE-stained, unstimulated cells (green subpopulation) mixed at day 4 with infected, stimulated, unstained cells (red subpopulation). Representative results are shown from one of 3 independent experiments, using cells from different donors.

The majority of HIV replication *in vivo* occurs in the microenvironment of primary lymphoid tissue, such as lymph nodes. It is probable that CD4 T lymphocytes become productively infected with HIV during the process of antigen-induced T cell activation and proliferation. In such a setting, persistent infection of CD4 T cells could result from the infection of cytokine activated cells that surround the immune response foci, which can be defined as “immunologic bystanders”. To investigate whether latently infected cells produced in our model were derived through a similar scenario, we designed an experimental approach to mimic our vision of such a biological environment. The experimental design (Fig 7A) involved staining a portion of freshly isolated CD4 T cells with CFSE, and maintaining these stained cells without stimulation for 4 days (condition 2). Another portion of CD4 T cells were not stained, but were infected and stimulated (condition 3) according to our standard *in vitro* protocol (Fig 1A). After 4 days of culture, the unstimulated and uninfected CFSE stained cells were mixed with the fully activated and infected cells at a ratio of 1:1 (condition 2+3). As a positive control, freshly isolated CFSE stained CD4 T cells were infected, stimulated, and cultured in parallel for 14 days (condition 1). To block HIV spread and superinfection, Indinavir was added to the cultures on day 7. Infection was monitored by flow cytometry for expression of intracellular Gag (ICp24/55). As shown in a representative experiment (Fig 7B), 20%– 30% of fully activated, infected cells expressed intracellular Gag at the peak of the infection on day 7 (condition 1, top row). Unstimulated CFSE-stained cells, cultured in the presence of fully activated infected cells (condition 2+3, bottom row), also exhibited intracellular Gag expression at day 7 in a small percentage of cells (1%), demonstrating that such non-dividing cells were indeed infected. Both the fully activated cells and the non-dividing cells lost intracellular Gag (ICp24/55) expression by day 13. At the end of co-culture, the cells were sorted into bright CFSE-positive (CFSE+; non-dividing, bystander cells) and CFSE-negative (CFSE**-**; fully activated, proliferating cells) subpopulations. Each recovered cell subset was analyzed for the quantity of integrated HIV by qPCR analysis and the frequency of replication-competent virus (IUPM) by terminal dilution co-culture assay. Results from 3 independent experiments (Table 3) showed that although the percentage of productively infected cells in the proliferating CFSE-negative (CFSE**-**) cell population was much greater than that in the non-dividing CFSE-positive (CFSE+) cell population (day 7, Fig 7B), the amount of integrated HIV DNA and replication-competent virus found at the end of culture were similar in both subpopulations (CFSE**-** and CFSE**+**), or sometimes even slightly greater in the CFSE^**+**^ bystander cell subset.

**Table 3:** Integrated HIV copies and cell-associated infectious units in non-dividing (CFSE^+^) vs dividing (CFSE^-^) subsets.

	Day 14 Cell Subset (CFSE staining)^a^	HIV_int_ / 500ng^b^	Infectious Units/10^6^ cells^c^
Donor 1	CFSE +	37,165	25,030
	CFSE -	15,360	25,030
Donor 2	CFSE +	7,515	817
	CFSE -	6,440	162
Donor 3	CFSE +	4,100	1,429
	CFSE -	6,305	817

^a^Results derived from experiments that used design outlined in Fig 7B (condition 2+3). Sorted cell subsets (CFSE+ and CFSE**-**) recovered from end of culture (day 14) were taken for: i) isolation of genomic DNA and qPCR determination of HIV integrant copies, and ii) restimulation and quantification of cell-associated infectious units (IUPM) by limiting dilution viral outgrowth assay (QVOA). Data obtained from 3 independent experiments, using cells from different donors.

^b^500 ng genomic DNA = 80,000 cell equivalents

^c^IUPM, infectious units per million cells

### Optimization of the bystander cell infection model to study establishment of HIV latency

To optimize the design described above, additional experiments were done to generate a greater proportion of infected cells with increased viability in a shorter time period. First, varying ratios of unstimulated/uninfected to stimulated/infected cell mixtures (1:1, 2:1, 4:1) were compared. At the end of a 14-day culture, a 4:1 mixture ratio produced greater numbers of infected resting CD4 T cells that contained approximately 2.5 fold more infectious cell units (IUPM) than did a 1:1 mixture ratio. Although the total number of HIV integrants per 500 ng genomic DNA (80,000 cell equivalents) in a 4:1 mix was only 20% of that seen in a 1:1 mix, the proportion of integrated provirus that was inducible was increased in the 4:1 mixture. This summarized data set was derived from 3 independent experiments, using cells isolated from different donors. Second, we determined whether the non-dividing bystander cells could be removed earlier from the co-culture mixture and still contain a significant amount of latent HIV infection. Because peak productive virus replication occurred consistently on day 7 of culture (Fig 2A), we reasoned that maximal transfer of infection to the bystander subpopulation probably occurred in parallel. When samples of resting bystander cells were removed by sorting on days 7, 9, 11, and 13 of culture, analysis of integrated viral DNA and IUPM demonstrated that cells taken at day 7 had a similar level of infection, as did cells taken at the later time points. In addition, the bystander cells isolated on day 7 were ≥94% viable in comparison to 40–50% viability of the cells isolated on day 13. Next, we determined that initial CFSE-staining of the infected, stimulated subpopulation (rather than staining of the resting bystander subset, as described above) could be used to effectively discriminate and sort unlabeled bystander cells from the co-culture mixture at day 7 (S2 Fig). This change in approach was taken in order to simplify any phenotypic analysis of the recovered bystander cells and to allow use of GFP reporter virus clones in future work. Lastly, we used BrdU incorporation (overnight) to examine the cellular activation state of the bystander cell population, following sorting at day 7. The percentage of BrdU-positive cells was 0.2–0.5% one day after sorting and fell to <0.1% after two and three days. In parallel, cell viability dropped from 88% to 76%, but the amount of total cell-associated HIV DNA and secondary reactivation of virus did not vary significantly over this period (S1 Table).

When cellular genomic DNA isolated from infected bystander cells, recovered at day 9 from the optimized culture design, was assayed for integrated provirus (Table 4), the quantity of HIV DNA ranged from 1,374–8,076 copies per 80,000 cell equivalents. These same bystander cells (recovered at day 9) were tested for their capacity to respond to TCR stimulation. Flow cytometry was used to analyze intracellular Gag expression, following 48 hr stimulation by beads coated with covalently-linked anti-CD3 + anti-CD28 antibodies (Human T-Activator DynaBeads) in the presence of the integrase inhibitor, raltegravir (0.5 μM) to block any induced replication from the presence of pre-integration species of HIV DNA (refer to Materials and Methods for complete details). These experiments demonstrated that the bystander cells contained inducible latent virus, ranging from 979 to 3,291 IUPM (Table 4). From these results, we determined that such bystander cells isolated from mixed co-culture at day 7, and held an additional 2 days without exogenous stimuli, provide an optimal *in vitro* model for mechanistic studies of HIV latency.

**Table 4:** Integrated HIV DNA, IUPM, and integrants/IU in latently infected bystander CD4 cells derived from the *in vitro* co-culture model.

Donor #	Integrated HIV DNA (copies/500ng)^a^	IUPM^b^	Integrants/IU^c^
**1**	3,211	3,170	13.4
**2**	8,076	2,700	39.5
**3**	6,027	2,433	32.7
**4**	1,374	1,864	9.7
**5**	3,561	3,231	14.5
**6**	1,931	979	26
**7**	5,279	3,291	21.2
**Average**	**4,208**	**2,524**	**22**
**SD**	**2,382**	**853**	**11**

**Fig 8:**
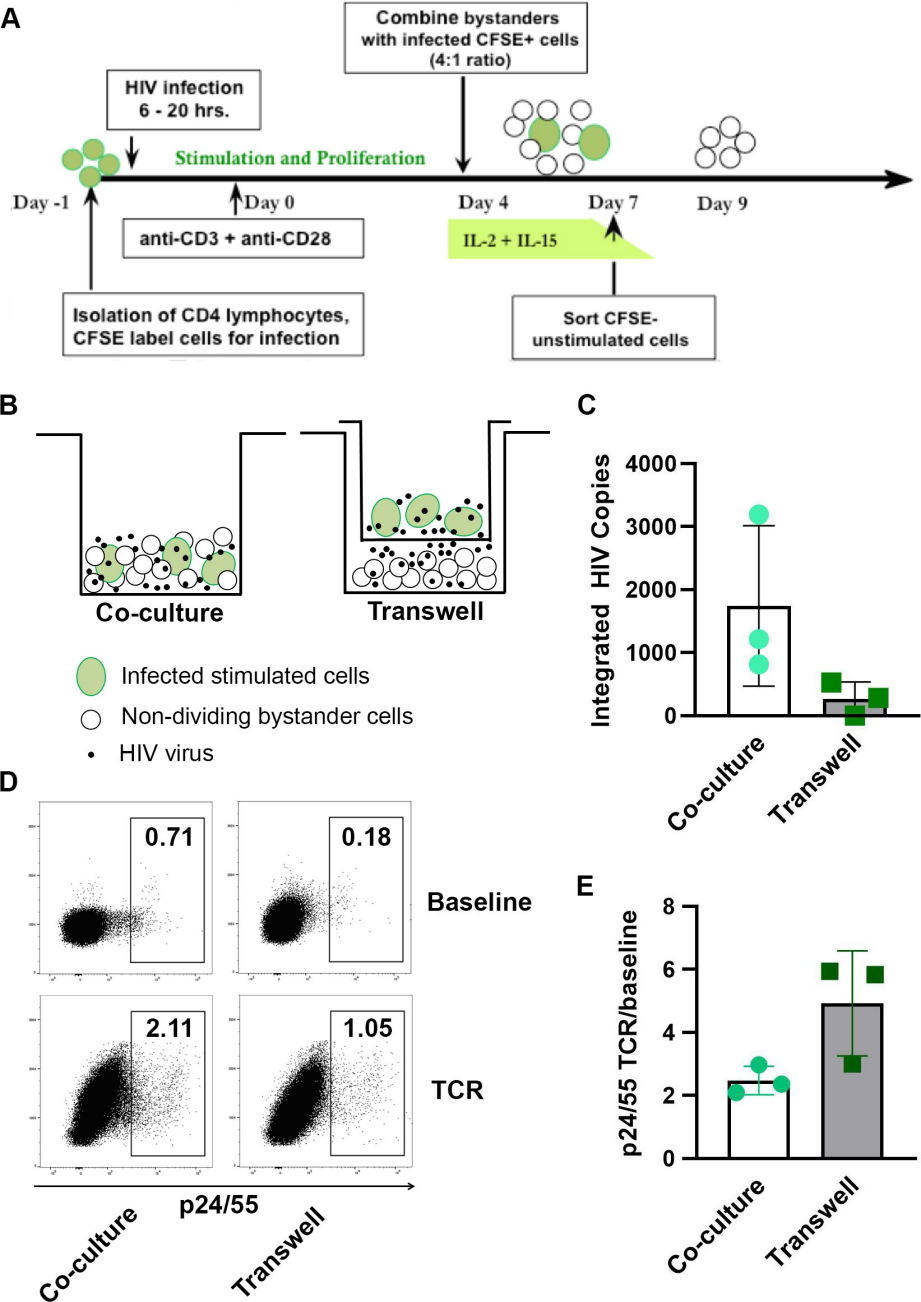
Optimized model of bystander cell infection and the importance of cell-to-cell contact. (A) Diagram of the optimized *in vitro* bystander cell model to generate latently infected primary CD4 T cells. A portion of isolated primary CD4 T lymphocytes were labeled with CFSE dye, held overnight, washed, and then acutely infected (6–20 hr) with NL4-3. Following infection, the cells were cultured in flat-bottom plates with immobilized anti-CD3 + anti-CD28 antibodies to stimulate cell proliferation and virus replication. Four days following activation, the infected CSFE-labeled cells were removed from the TCR stimulus and mixed with unstimulated, uninfected autologous CD4 T cells (unlabeled); and the cell mixture, cultured in fresh medium containing exogenous rIL-2 and rIL-15 for 3 days. On day 7 of culture, the non-dividing CFSE-negative subpopulation (“bystander cells”) was isolated by FACS. The recovered resting cells, which carry non-productive latent HIV infection, were cultured in fresh medium without cytokines for 2–3 days, before use in subsequent experiments. (B) Schematic representation of the transwell membrane culture system. (C) HIV integration was quantified by ddPCR in the target bystander cells, following infection in transwell cultures or direct co-culture cell mixtures. Results shown as HIV integrants per 80,000 cell equivalents. (D) Representative example of flow cytometry gating strategy to quantify percentages of cultured cells that expressed intracellular Gag (p24/25), following TCR stimulation for 48 hr. Note that percentage of Gag-positive cells, following establishment of latent infection, was lower in the transwell cultures than in the direct mixed cell co-cultures. (E) Fold increases (TCR stimulated / baseline) in the percentages of cells expressing intracellular Gag (ICp24/25), following 48 hr TCR stimulation. Results for C. and E. depict the mean values ± SD (error bars) of 3 experiments, using cells from different donors.

Experimental design based on schema outlined in Fig 8A. On Day 9, cell aliquots were taken for: i) isolation of genomic DNA and qPCR determination of HIV integrant copies, and ii) re-stimulation using anti-CD3 + anti-CD28 coated beads in the presence of HIV integrase inhibitor, raltegravir. Following 48 hrs of stimulation, cell-associated infectious units were determined using flow cytometry analysis of intracellular Gag expression. Results obtained from 7 independent experiments, using cells from different donors.

^a^500 ng genomic DNA = 80,000 cell equivalents

^b^IUPM, infectious units per million cells

^c^Integrants per infectious unit (IU) were obtained for each experiment by calculating the integrated HIV copies per cell (i.e. copies per 500 ng DNA divided by 80,000 cell equivalents) followed by division with the sample associated IUPM. The average value (Mean) was then derived from these individual ratios.

The non-dividing “bystander” CD4 T cells, obtained via this co-culture model (Fig 8A), contained levels of integrated and infectious HIV very similar to those that we found previously in latently infected cells, derived from 14-day TCR-stimulated CD4 T cell cultures, which had undergone acute productive infection and cell division. The latently infected primary CD4 T cells, generated by our method, possessed the same phenotypic characteristics of infected T lymphocytes that comprise the major latent HIV reservoir *in vivo*. However, this *in vitro* cell model has the benefit of producing numbers of HIV integrants and associated infectious units per cell (Table 4) that are significantly greater than the proportion of latently infected CD4 T cells, which can be obtained from persons living with HIV.

### Role of cell-to-cell contact in establishing HIV infection of “bystander” cells

Next, we examined the role of direct cell-to-cell contact for establishment of latent HIV infection in our bystander cell model. To evaluate this, our optimal co-culture model (Fig 8A) was used to set-up a parallel infection design in which half of the microcultures contained resting bystander cells that were physically separated from the stimulated, productively infected cells by a 0.4 μM transwell membrane (Fig 8B). Agosto and colleagues had previously demonstrated that virus particles can freely pass through such a barrier, although cells are prevented from doing so [27]. Non-dividing bystander cells from the co-culture design were separated from stimulated, productively infected cells on day 7, using fluorescence activated cell sorting (FACS). In parallel, the resting bystander cells, separated from productively infected cells by the transwell membrane were collected from culture; and the absence of productively infected cells in this fraction was confirmed by flow cytometry analysis, using a small aliquot of cells. The resting cells recovered from both co-culture conditions were cultured for an additional 3 days. A portion of cells from each condition was used to assess the frequency of viral integration events, using droplet digital PCR (ddPCR). HIV integration was detected in three independent co-culture experiments (direct cell-to-cell contact); however, integration events from transwell-based infections were not detected in cells from one donor and occurred at lower frequencies in cells from the other two donors (Fig 8C). A second portion of recovered bystander cells, harvested from each co-culture condition (cell-to-cell contact *vs* transwell membrane separation) was used to compare their capacity to respond to TCR induction of productive viral replication. Cells were activated by stimulation with anti-CD3 + anti-CD28-coated beads for 48 hr in the presence of HIV integrase inhibitor, raltegravir. Induced expression of intracellular Gag (ICp24/55) was measured by flow cytometry analysis. While expression of induced Gag was less in cells that were infected in transwell culture compared to cells infected by direct cell-to-cell contact (Fig 8D), the fold increase in intracellular Gag-expressing cells, following TCR stimulation was greater for latency established in transwell culture (Fig 8E). Taken together, these results support the hypothesis that cell-to-cell virus transmission is more efficient than cell-free infection in establishing HIV latency, but the resulting latent cell pool may be more resistant to virus induction [27].

## Discussion

The mechanisms involved in the establishment of HIV latency in CD4 T cells have not yet been clearly defined. In the studies described herein, we have used a unique *in vitro* primary T cell model to investigate cellular activation and proliferation events that foster the establishment of HIV latency in CD4 T lymphocytes. We show that CD4 T cells, exposed to virus prior to induction of cell activation, are more likely to develop stable latent infection. Most other *in vitro* studies of HIV infection have exposed pre-stimulated T cells to virus to quickly establish acute infection and abundant virion production. However, HIV infection of proliferating T cells results mainly in apoptotic cell death [43]; while, unstimulated cells appear to survive infection more readily [44]. In our model, infection of resting T cells, followed by the addition of a potent cell stimulus, drives virus integration and generation of the greatest number of latently infected cells that survive to 14 days in culture.

These results demonstrate that HIV latency is established very early within a heterogeneous population of primary CD4 T cells, which are undergoing varying degrees of cell activation in the presence of infectious virus. Latency is established preferentially in non-dividing T cells, that exhibit suboptimal levels of activation, as shown by varied expression of antigens associated with activation and exhaustion, and are more likely to survive during the early stages of infection. In addition, no evidence could be found in our *in vitro* model system for the viral protein, Vpr to induce cell cycle arrest [40] or influence the development of latent infection in the non-dividing T cell subpopulation.

Several publications have reported that a significant portion of the CD4 T lymphocyte pool, which comprises the HIV latent reservoir *in vivo*, is established throughout various tissue compartments very early after the detection of acute HIV infection in patients [5, 8]. Given the inherent restrictions of any cell culture system to mimic biologic events occurring in the whole organism, our primary T cell model of HIV latency appears to reflect accurately at least one mechanistic component of HIV latency *in vivo*—the early establishment of latent/persistent infection in non-dividing T cells, in a shared environment of simultaneous, ongoing active viral replication. The majority of the long-lived reservoir in persons living with HIV on combination antiretroviral therapy (cART) is likely seeded shortly before therapy initiation [45, 46]. Therefore, the mechanistic underpinnings of reservoir establishment described by our studies would be most relevant for patients in resource-rich settings where early initiation of cART is a standard of care. Our experimental observations are consistent also with previous reports that show most infected cells in humans and macaques do not have an activated phenotype [19], and that naïve CD4 T cells can contain latent infection *in vivo* [47–50].

Three main hypotheses have been proposed to explain development of HIV latency in primary CD4 T cells [51]: i) an activated, proliferating cell becomes infected and reverts back to a resting state [52–54]; ii) an activated cell becomes infected during the narrow window of time when it is returning to quiescence [33, 52]; or iii) infection is established directly in a resting CD4 T cell [17, 24, 25, 55, 56]. Of most relevance to natural HIV infection *in vivo*, resting CD4 T lymphocytes, containing both viral DNA and RNA, have been found in animal models and infected humans; and such infected populations of resting T cells exhibit a significantly longer half-life than that of activated T cells [19]. From these findings, investigators have hypothesized that lymphokines present in the immune tissue milieu may provide a permissive environment for viral infection and spread in non-dividing T cells. Because gamma-chain cytokines may promote cell proliferation in the absence of virus activation, this environment can facilitate propagation of already established reservoirs [57]. Importantly, infection of resting CD4 T cells and establishment of HIV latency can be enhanced through co-culture with antigen-presenting cells, infected or uninfected [58, 59], or virus-producing endothelial cells [60, 61]. Additionally, it has been demonstrated that HIV latency can also be established in resting CD4 T cells via cell-to-cell contacts with infected proliferating T cells [27].

Results from our own studies are consistent with the idea that cell-to-cell interaction is an important contributor to HIV infection and establishment of latency in non-dividing CD4 T cells. The comparison of total HIV provirus load and its inducible replication-competent portion in cells that have undergone different amounts of cell cycle division suggests strongly that infection of sub-optimally activated, non-proliferating T cells is an important component in the development of HIV latency. Cell-to-cell transmission and homeostatic cell proliferation likely represent two complementary mechanisms by which the latent viral reservoir can be spread in tissue microenvironments. Our own data argue against a significant contribution to the latently infected T cell pool through infection of fully activated cells, during either proliferation or reversion back to a resting state. Such results are consistent with an earlier study by Duverger and colleagues who proposed that the generation of latent infection is independent of the requirement for a reduction in the cellular activation state [62].

A number of human primary T cell models have been developed to mimic various biologic aspects that are characteristic of HIV latency *in vivo*; each of these *in vitro* systems offers certain advantages and disadvantages to study mechanisms of viral latency (reviewed in: [53, 63–65]). Unfortunately, all these model systems together have not been able to identify a central unifying theme for proposed mechanistic pathways, controlling HIV latency [66]. It is quite plausible that subtle differences in the design of these cell model systems make significant impacts on how latency is established initially and by which molecular mechanisms have overriding control of viral maintenance and reactivation [10]. The approaches used to establish latent infection vary widely among such models, including the expansion of proliferating infected cells and their return to quiescence (resultant uniform central memory phenotype) [67, 68]; and the direct infection of T cells that have not undergone cell division in culture (resultant mixture of all maturation phenotypes seen *in vivo*) [25, 26, 58, 69]. The cell model, which we have derived and is described herein, produces latent HIV infection via cell-to-cell virus transmission in culture, within multiple subpopulations of primary CD4 T cells that include all the known major maturation phenotypes, and may represent more accurately the potential mechanism(s) by which latent infection develops *in vivo*. Infection and establishment of viral latency in the naïve CD4 T cell subset is, perhaps, the most important and unique aspect of our *in vitro* cell model. Although infected naïve T cells have historically been considered minor contributors to the latent HIV reservoir *in vivo*, findings from recent published reports have demonstrated that the level of naïve T cell infection is a predictor of viral reservoir size and diversity [70, 71]. Therefore, we propose that our primary T cell model of HIV latency represents a valuable asset to the studies of potential mechanisms, which are involved in the establishment and maintenance of viral latency.

We believe further investigations are warranted, in which a spectrum of primary T cell model systems are employed and compared to gain a better understanding of whether the route taken to establish the latent viral state directly influences the degree to which specific molecular mechanisms control the maintenance and reversal of HIV latency. Moreover, several recent reports have highlighted the importance of examining multiple cell types in mechanistic studies of HIV latency [72, 73], giving support to the use of more unbiased approaches for studies of viral latency in phenotypically heterogeneous populations of primary CD4 T lymphocytes. In addition, the possible existence of such inter-dependent relationships in the establishment of viral latency could confound the ongoing search for relevant cell biomarkers of latent HIV infection, which are needed for development of new treatment strategies to selectively target latently infected cells. The route by which latent infection is established should be considered when primary cell models are chosen to validate candidate biomarkers. If critical variables are inadvertently excluded from the testing paradigm, the results may not capture valuable information about the range of cell-associated components, which are important to the latent viral state. Given what is known currently about HIV latency, it is highly probable that a complex panel of cell biomarkers will be required to identify and target the various cell components, which comprise the majority of the latently infected HIV reservoir *in vivo*.

A limitation of our present studies is the use of a CXCR4-tropic lab-adapted viral strain (NL4-3), which is highly replication competent and pathogenic in proliferating T cells, and is not representative of the CCR5-tropic virus strains that are predominantly found in HIV-infected patients. Unfortunately, this is a common limitation of the vast majority of available current *in vitro* models of latent infection in CD4 T cells. Development of easily accessible cell model systems that use CCR5-tropic wild type virus clones is crucial to the advancement of this area in HIV research. And, it is necessary for biologically relevant evaluation of how and to what extent viral tropism may affect the mechanisms of establishment and maintenance of viral latency in different CD4 T cell subsets *in vivo*. Because the CCR5 receptor is expressed predominantly on memory cells, and its level of expression is highly sensitive to the form of T cell activation used during *in vitro* culture [74], it is extremely likely that had we been able to use a CCR5-tropic HIV clone to establish infection in our model of latency, the results obtained may have reflected quite different ratios for the prevalence of latent infection in CD4 T cells with minimal activation and cell proliferation.

Another potential criticism of this report concerns the methodology that we use routinely to quantify cell-associated integrated HIV DNA in our *in vitro* model systems (please refer to Materials and Methods for complete details). In our 14-day culture model, we did not assess latently infected samples for total HIV DNA, but rather focused on the high molecular weight (23 kb) species that is recovered at the end of culture, after 7–9 days in the presence of antiretroviral drugs and when ≥98% of the T cells have returned to a resting state. Other previously published reports, using viral latency models have documented the contribution of unintegrated HIV DNA to viral RNA and even protein production [20, 75, 76]; however, these systems contained unique culture aspects very different from our 14-day latency model and used direct infection of resting cells. That being said, we recognize that T cells from our mixed co-culture “bystander” model of latency probably do contain linear unintegrated viral DNA species when recovered by cell sorting (FACS) at day 7 (refer to Fig 8A). And, that is precisely why raltegravir, integrase inhibitor was added to cultures 2–3 days after cell isolation, at the same time as TCR stimulation, to block induced replication from the presence of pre-integration viral DNA species. The bystander cell model was designed specifically for evaluation of latency reversing agents [64]; our current study has not addressed the possible contribution of unintegrated viral DNA to the detected responses induced by TCR stimulation. Future studies will need to be designed to evaluate any contributions of unintegrated HIV DNA to the virus reactivation responses seen in this model. If such contributions are substantial, use of the T cell model could potentially benefit studies to dissect mechanisms of both post- and pre-integration latency.

Because the main obstacle to treatment induced “functional eradication” of HIV infection is viral latency, recent efforts in HIV therapy development have turned towards strategies to target the viral reservoirs and interfere with the control of latent infection. However, before rational effective therapeutic approaches can be designed, a more thorough understanding of the basic biologic mechanisms underlying the establishment and maintenance of persistent, nonproductive HIV infection is needed. The results from our current studies implicate the likely importance of direct infection of sub-optimally activated T cells in establishment of latently infected reservoirs *in vivo*, especially in CD4 T lymphocytes that surround productive viral foci within immune tissue microenvironments.

## Materials and methods

### Primary CD4 T lymphocyte cultures

Peripheral blood was collected from HIV-seronegative donors into heparin sodium–containing syringes, according to the protocol approved by the institutional review board of the University of California San Diego Human Research Protection Program. Study participants provided written informed consent. Total CD4 T lymphocytes were isolated by negative selection from whole blood buffy coats using the RosetteSep cell separation procedure (StemCell Technologies), followed by density gradient centrifugation with Histopaque 1077 (Sigma-Aldrich) or Lymphoprep Cell Separation Medium (Accurate Chemical). Resulting cell preparations were monitored for viability by trypan blue dye exclusion and for phenotype purity by flow cytometry analysis; they were routinely >95% viable and positive for CD4 expression. The isolated cells were maintained in RPMI 1640 medium (Gibco-Invitrogen) supplemented with 2 mM L-glutamine, 100 U/mL penicillin, 100 μg/mL streptomycin, and 5% (vol/vol) human AB serum (Omega Scientific; RPMI/5%HAB). Initially, 2.5–5 x 10^6^ cells per 2 mL medium were distributed into each well of a 6-well, flat-bottom plate. To induce cell stimulation, a non-tissue culture treated plate that was pre-coated with goat anti-mouse IgG plus anti-CD3 (0.03 μg/mL) and anti-CD28 (0.2 μg/mL) monoclonal antibodies (BD Biosciences) was used, as previously described [36]. After four days, cells were removed from the antibody-coated plates, washed and transferred into 6-well, tissue culture treated plates. For long-term culture out to 14 days (Fig 1A), the cells were maintained at a concentration of 3–5 x 10^6^ cells per well in RPMI/5%HAB medium for the remainder of the culture period (13–14 days). A mixture of cytokines was used to optimize cell proliferation and survival: IL-2 (5 U/mL final, NIH AIDS Research and Reference Reagent Program) was added on day 4; IL-15 (10 ng/mL final, R&D Systems) was added on days 4 and 7; and IFNβ (833 U/mL final, PBL InterferonSource) was added on days 10 and 12 of culture to reduce activation-induced T cell death [32]. Where noted, reverse transcriptase inhibitor, Nevirapine (10 μM) or protease inhibitor, Indinavir (1 μM) was added to infected cultures on day 5 or day 7, respectively, and maintained throughout the remainder of the culture period. Dependent on the mechanism of action, addition of the antiretroviral drugs was timed to maximize potential HIV integration events prior to drug-induced interferences in viral replication and spread. As a reverse transriptase inhibitor, Nevirapine that was added on day 5 should have blocked the replication of new incoming virus but not interfered with integration of viral DNA species, already fully formed. Indinavir, a protease inhibitor would have affected viral replication post-integration at a much later stage in the cycle, by interfering with viral budding to produce noninfectious virion particles. Therefore, day 7 was selected.

For the establishment of co-cultures to produce latently-infected resting bystander cells (Fig 8A), a portion of the freshly isolated CD4 T cells was stained with CFSE dye (10 μM, Molecular Probes); held in culture overnight; washed and infected with NL4-3; and then cultured in plates with immobilized anti-CD3 + anti-CD28 antibodies (as described above) for 4 days to induce cell proliferation and productive viral replication. Another cell aliquot (unstained and uninfected) was maintained in parallel without stimulation. At this point, the CFSE-stained, infected and proliferating cells were removed from the anti-CD3/anti-CD28 stimulus and mixed with the autologous, unstimulated resting CD4 T cells at a ratio of 1:4. In most experiments, the cell mixture was distributed into flat-bottom wells of 6-well, tissue culture treated plates at a concentration of 5 x 10^6^ cells per 3 mL medium (RPMI/5%HAB) per well in the presence of exogenous rIL-2 (5 U/mL) and rIL-15 (10 ng/mL). Antiretroviral treatment was not added. In experiments using transwell cultures, 4 parts of the unstimulated resting cells in the presence of IL-2 and IL-15 were added to the bottom of wells in a 24-well tissue culture treated polysterene plate with polycarbonate membrane inserts (6.5 mm diameter, 0.4 μm pore, Corning Costar); and one part of CFSE-stained, infected and proliferating cells in the presence of IL-2 and IL-15 were added to the culture volume above the transwell insert. The regular 4:1 (resting uninfected bystander: proliferating infected) cell mixtures in a total volume of 1 ml were added in parallel to the same plate in wells that lacked a transwell membrane insert. After 3 days of co-culture, the cells were collected, washed, and counted, using the trypan blue dye exclusion method. The non-dividing CFSE-negative subpopulation (“bystander cells”) was isolated by flow cytometry cell sorting, using a MoFlo XDP instrument (Beckman-Coulter) or BD FACS Aria II sorter. In transwell culture, the resting CFSE-negative bystander cells were collected from the bottom section of the well; and the absence of productively infected cells in this fraction was confirmed by flow cytometry analysis, using a small cell aliquot. The recovered resting cells, carrying non-productive HIV infection, were cultured in fresh complete medium (RPMI/5%HAB) for 2–3 additional days, before being used in subsequent experiments.

### Cell staining and flow cytometry analysis

The purity of CD4 T lymphocyte preparations was monitored by staining with FITC-conjugated anti-CD4 and anti-CD19 antibodies, and PE-conjugated anti-CD16 and anti-CD8 antibodies (BD Biosciences). Anti-CD25 and anti-HLA-DR antibodies conjugated to APC or PE, respectively (BD Biosciences) were used to measure expression of activation markers. Cell surface staining was performed for 30 min at 4˚C with cell aliquots containing 0.5 x 10^6^ to 1 x 10^6^ cells. For DNA/cell cycle analysis, cells were labeled either in a 2 hr pulse (activated condition) or overnight (resting condition) with bromodeoxyuridine (BrdU) provided in the BD/Pharmingen BrdU Flow Kit. Cell aliquots were then fixed, permeabilized, and stained according to the manufacturer’s instructions. Addition of 7-amino-actinomycin D (7-AAD; Molecular Probes-Invitrogen) was used to assess DNA content for cell cycle distribution. Fifty thousand cell events were analyzed (BD FACSCanto II, DiVa software 2.1). To track cell proliferation, 10 x 10^6^ cells were labeled with 10 μM CFSE (Molecular Probes) in 0.1% bovine serum albumin supplemented Dulbecco’s phosphate buffered saline, calcium- and magnesium-free (PBS; Mediatech) for 5 min at room temperature, then quenched with 10 mL of cold RPMI medium with 10% HAB serum, placed on ice for 5 minutes, and washed with 5 mL warm RPMI/5% HAB medium. Labeled cell samples were analyzed (BD FACSCanto II, DiVa software 2.1) and/or sorted (MoFlo Legacy or XDP instrument, Beckman-Coulter). For intracellular Gag (ICp24/55) analysis, cells were washed in PBS, fixed and permeabilized with Cytofix/Cytoperm Buffer (BD Biosciences), using a modification of a published method [77]. After washing with Perm/Wash Buffer (BD Biosciences), cells were incubated for 30 min with KC57-RD1, PE-conjugated antibody (Beckman-Coulter). Cells were washed again with Perm/Wash buffer and stored in 0.5% formaldehyde at 4°C, until flow cytometry acquisition and analysis was performed (BD FACSCanto II, DiVa software 2.1). For analysis of maturation phenotype subsets in CFSE labeled and cultured cells, 5 x 10^6^ cells were stained with the viability dye Live/Dead Aqua (Molecular Probes/Invitrogen), and surface stained with APC-conjugated anti-CD45RA, APC-H7-conjugated anti-CD27, PE-conjugated anti-CD25, Per-CP-Cy5.5-conjugated anti-CD28, PE-Cy7-conjugated anti-CD38, and V450-conjugated anti-CD4 (BD Biosciences), as described above for analysis of CD4 T cell purity. For these experiments, ≥5,000 viable CFSE-bright cell (non-proliferating) events were acquired for T cell phenotype determinations. For experiments to characterize the activation and exhaustion-associated status of CD4 T cells that did or did not divide in response to TCR stimulation, 3–5 x 10^4^ cells were stained with Aqua Live/Dead, APC-conjugated anti-CD4, PerCP-Cy5.5-conjugated anti-CD69, PE-Cy7-conjugated anti-CD25, AF700-conjugated anti-CD38, PE-conjugated anti-PD-1, and BV605-conjugated anti-TIGIT (BD Biosciences). Unstimulated cells were used as controls to set positive/negative gating regions.

### Virus infection

Infectious virus stocks of the NL4-3 clone of HIV-1 [78] were prepared by transfecting plasmid DNA into the CEM T lymphoblastoid cell line with Lipofectin (Invitrogen), as previously described [21]. Virus preparations were quantified for infectivity by limiting dilution infection of CEM cells, or via the P4R5 MAGI blue cell assay [79]. Aliquots of 4–6 x 10^6^ CD4 T lymphocytes were incubated with 0.5 mL of NL4-3 virus for 6–20 hrs at 37°C at a multiplicity of infection (MOI) of 0.01 TCID_50_ (CEM cells) or 0.01 IU (MAGI assay). After infection, excess virus was removed by extensive washing with PBS containing 2%HAB serum. Cells were cultured, as described above. Productive HIV replication was determined by measuring either the amount of soluble p24 antigen released into culture supernatants using ELISA (Abbott) or the number of cells expressing intracellular Gag protein using flow cytometry. Plasmid DNA containing the vpr mutant virus (pNL4-3-VprX) was a generous gift from Dr. Vicente Planelles. It was originally generated from pNL4-3 by digestion, end filling and religation of the EcoRI site in the vpr coding sequence, which results in a frame shift after I63 and a premature stop codon after amino acid 79 [41]. The plasmid was verified by restriction digest and Sanger sequencing of the Vpr coding region. Infectious stock of the vpr mutant virus was prepared by transfecting plasmid DNA into the P4R5 MAGI cell line and quantified by the MAGI blue cell assay [79]. Cells were infected with wild type NL4-3 and the isogenic vpr mutant clone at the same MOI and using equal final volumes of virus stock plus medium.

### Quantification of integrated HIV DNA

Infected cells and uninfected cell controls were collected at sequential time-points during culture and stored frozen in dry pellet aliquots of 1–2 x 10^6^ cells. In batched analysis, cell pellets were thawed and resuspended in 200 μL Dulbecco’s PBS. DNA was prepared using the Qiagen Blood and Cell Culture DNA Mini or Micro kits, according to the manufacturer’s instructions (Qiagen). Cellular genomic DNA was physically separated from circular and linear unintegrated viral DNA forms, using a modification, as detailed herein, of a previously published method [80]. Up to 2 μg of sample DNA were run on a 0.5% low melt agarose (Genessee Scientific, Inc.) TAE (Invitrogen) gel, at 60 volts for 4 hours. DNA bands ≥ 23 kbp in size were excised with Gene Capsule devices (G-Biosciences), melted at 70°C for 5 min, cooled to 42°C for 5 min, then digested with beta-agarase (Fermentas) at 42°C for 30 min. Samples were extracted once with one volume of equilibrated phenol (USB), once with 25:24:1 phenol:chloroform:isoamyl alcohol (USB), and twice with chloroform (Sigma Chemical Company). DNA was precipitated with 20 μg glycogen (Fermentas), 0.2 volumes 3M sodium acetate, pH 5.2 (Teknova), and 2 volumes absolute ethanol at -20°C overnight or -80°C for at least 30 min. The extracted DNA was pelleted and washed twice with 70% ethanol, air dried briefly, and resuspended in 25 μL 10mM Tris, pH 8. In some experiments, Zymoclean large fragment DNA recovery kit (Zymoresearch) was used instead for purification of DNA from gel. DNA samples were assayed by TaqMan real-time qPCR or ddPCR for cellular genomic input using RNAseP (*RPP30*) primers and VIC- or HEX-labeled probe (Applied Biosystems, Inc.), multiplexed with HIV-1 pol or gag primers and FAM-labeled probe, as designed originally by Althaus, et al. [81] and Michael et al. [82], respectively, and synthesized by Integrated DNA Technologies:

pol forward: mf299 5’-GCACTTTAAATTTTCCCATTAGTCCTA-3’,

pol reverse: mf302 5’-CAAATTTCTACTAAT GCTTTTATTTTTTC-3’,

pol probe: mf348 5’-/56-FAM/AAGCCAGGAATGGATGGCC/3BHQ-1/-3’,

gag forward: SK462 5’-AGTTGGAGGACATCAAGCAGCCATGCAAAT-3’,

gag reverse: SK431 5’-TGCTATGTCAGTTCCCCTTGGTTCTCT-3’,

gag probe:

SK102 5’-/56-FAM/AGACCATCA/ZEN/ATGAGGAAGCTGCAGAATGGGAT/IBFQ/-3’.

2LTR circles were quantified using the following primers [83]:

forward: 5’-AACTAGGGAACCCACTGCTTAAG-3’,

reverse: 5’-TCCACAGATCAAGGATATCTTGTC-3’,

probe: 5’-/56-FAM/ACACTACTTGAAGCACTCAAGGCAAGCTTT/3BHQ-1/-3’.

Standard curves for real time qPCR quantification were generated with: genomic DNA from human PBMC of healthy controls (RNaseP); linearized pNL-EGFP plasmid (pol), diluted into a background of 1 μg of herring sperm DNA (Promega); and pG2LTR plasmid [84] in 200 ng of uninfected CD4 T cell genomic DNA (2LTR circle). HIV-1 pol or gag copy numbers were corrected for 2LTR circle co-purification, adjusted for the amount of input DNA, and expressed as copies/500 ng DNA, which equals 80,000 primary T cell equivalents.

The levels of proviral DNA (pol or gag detection) found within the recovered high-molecular-weight genomic DNA bands (HMW; ≥ 23 kbp) was determined for each experiment (data not shown) and routinely demonstrated ≥ 98% purity with ≤ 2% 2LTR circle contamination. Our method, using a DNA size selection approach is similar to, and based on the same principles of, the BluePippin (Sage Science) gel electrophoresis platform, reported by others [85]. The results for the quality of data, obtained by our described method are consistent with the findings of integrated HIV quantification in the other report, using cell samples from HIV-infected patients on suppressive cART [85].

### Quantification of HIV RNA transcripts

Based on a published approach [34] specific TaqMan primer and probe sets were designed to target unspliced, multiply-spliced, Nef-encoding, Tat-encoding, and singly-spliced Env-encoding species of HIV RNA (S2 Table). The amplification products (amplicons) spanned the splice donor (SD) to splice acceptor (SA) sites unique to the RNA species of interest, with either the primers or probe annealing across the splice junction (S3 Fig). Using RNA extracted from NL4-3 virion stocks (unspliced), and NL4-3 infected primary CD4 T cells (multiply spliced, Nef, Tat) or SupT1 cells (Env), cDNA amplicons were generated for each of the selected HIV splice variants. The cDNA products were cloned into plasmids using a TOPO^®^-cloning vector system. Bacterial transformations were performed to screen clones for the inserted amplicons of interest, and midi-preparations were made. Plasmids carrying the identified inserts were isolated, purified, and sequenced to confirm identity and proper orientation. The primer and probe sets were then tested for sensitivity and specificity of target sequence detection (S4 Fig). Sense-oriented RNA standards were *in vitro*-transcribed using the plasmid T7 promoter. The RNA standards were then DNase-treated, column purified, and quantified by spectrophotometry. The presence of a single band of the appropriate size was confirmed for each RNA amplicon standard, by denaturing agarose gel electrophoresis. These RNA standards were diluted into a constant background of extracted RNA from uninfected CEM cells and used for the quantification of absolute copy numbers of each HIV RNA splice variant present in experimental samples.

Primary CD4 T cells were harvested from culture at sequential times following infection and stimulation. Total cellular RNA was extracted (Qiagen RNAeasy kit) and RT-qPCR was performed with the samples, uninfected T cell RNA, and RNA standard controls to quantify each of the targeted HIV mRNA species (normalized to 25 ng input RNA). Up to 1 μg RNA was isolated, reverse transcribed by random hexamer priming, and analyzed by qPCR. Primers directed at unspliced RNA (*gag*), multiply-spliced RNA (ms), singly-spliced RNA (*env*), and the most abundant variants of *nef* and *tat* [85] were used at concentrations of 300 nM (ms and env) or 400 nM (unspliced, nef and tat) for each primer and 250 nM of each probe (S2 Table) in each reaction mixture. All probes were 5’-FAM, 3’-Black Hole Quencher labeled (Integrated DNA Technologies). Because the unspliced mRNA target did not span an intron, control cDNA reactions, lacking reverse transcriptase, were performed and amplified in parallel to control for any residual viral DNA in the samples. Standard curves for each transcript were generated by serially diluting the reverse transcribed, cloned RNA into uninfected cellular cDNA, for a total of 25 ng input RNA per reaction. Sensitivity of detection was routinely 10 copies per reaction for all targets.

### Quantification of infectious units per million cells (IUPM)

Two methods were used to determine infectious units in latently infected cells recovered from culture: terminal dilution co-culture assay, IUPM (Tables 1 and 3), and flow cytometry analysis of induced intracellular Gag (ICp24/55) expression, cell-associated IU (Table 4). In the terminal dilution assay, cells recovered from culture were washed and resuspended in RPMI/5%HAB medium at 5 x 10^5^ cells per mL; 200 μL of cell suspension were distributed into duplicate wells of a 96-well flat-bottom plate, coated with anti-CD3 + anti-CD28 antibodies, as described above. Autologous uninfected cells from the same donor were used to dilute and amplify the detection of reactivated virus. Five-fold serial dilutions were made in duplicate into a constant background of 10^5^ uninfected cells. Unstimulated controls were derived from similar limiting dilution co-cultures that were distributed into uncoated, tissue-culture treated plates. The microculture plates were incubated for 7 days at 37˚C in 5% CO_2_ in air. Supernate (150 μL) from each duplicate well was collected and analyzed for level of soluble p24 by ELISA. Wells with p24 values >50 pg/mL were scored as positive. Infected cell frequencies were determined by the maximum likelihood method [86] and were expressed as infectious units per million CD4 T cells (IUPM). Flow cytometry determination of cell-associated infectious units (IU) used the staining method described above with the KC57-RD1 antibody, to detect intracellular Gag (ICp24/55) expression. Recovered infected cells were washed and cultured with anti-CD3 + anti-CD28 coated beads at a 1:1 bead:cell ratio (Human T-Activator DynaBeads, ThermoFisher Scientific) for 48 hr in complete RPMI medium in the presence of HIV integrase inhibitor, raltegravir (0.5 μM) to block any induced replication from the presence of pre-integration species of HIV DNA. An uninfected cell control was stimulated and cultured in parallel. Following induction, cells from both conditions were stained, fixed, and 50,000 cell events per sample were analyzed (BD FACSCanto II). Enumeration of cells, expressing intracellular Gag, was determined by gating on the bright positive cell subset in the infected cell sample and subtracting any positive events detected in the uninfected, stained cell control sample, using the same cursor setting (background staining).

## Supporting information

S1 Table Characterization of infected bystander cells held 1–3 days after sorting. For the first three variables, results are given as the mean ± SD from 4 experiments, using cells from different donors. Cell restimulation data, averaged from 2 experiments. HIV DNA = total cell associated. Induction of productive virus replication (p24, pg/ml), at 7 days after a secondary stimulation was analyzed by ELISA.
(DOCX)

S2 Table Primer and probe sequences for HIV RNA quantification. Primer and probe sequences used in the TaqMan qRT-PCR assay, for each of the five listed HIV transcript species. All probes were 5’ FAM and 3’ Black Hole Quencher conjugated. All targeted cDNA sequences were derived from the genomic sequence of the NL4-3 viral clone (Gen-Bank accession #M19921.1).
(DOCX)

S1 Fig Vpr does not affect activation and exhaustion status of CD4 T cells regardless of number of cell divisions in response to TCR stimulus. Aliquots of cells were stained with CFSE dye and infected in parallel with NL4-3 (wt) and vpr mutant (vpr**-**) virus (MOI 0.01 IU, 0.5 ml volume). Cells were activated on plates pre-coated with goat anti-mouse IgG plus anti-CD3 + anti-CD28 antibodies for 4 days. At this point, cells were removed from stimulation, resuspended in culture medium supplemented with IL-2 and IL-15, and transferred to new microculture plates. Samples were collected daily on days 4–7 for flow cytometry evaluation of cell surface antigen markers. Throughout the time course, wild type virus and vpr mutant were compared for expression of: (A) Activation markers (CD69, CD25, CD38); (B) Exhaustion-associated markers (PD-1 and TIGIT); (C) Surface expression of CD4 receptor.
(TIF)

S2 Fig Gating strategy to prevent contamination of sorted resting CFSE- cells with activated productively infected cells that lose CFSE as the result of cell division. Gating regions were set to exclude any cells from the activated productively infected sample from the CFSE^**-**^ population region. Despite potential loss of a small portion of resting CFSE^**-**^ cells in some experiments (e.g. Donor 1), contamination with productively infected cells is very negligible to non-existent.
(TIF)

S3 Fig Design of primers and probes for qRT-PCR. RNA specific Taqman amplicons are depicted by the yellow boxes. Primers or probes anneal across the splice donor (SD) and splice acceptor (SA) junctions.
(TIF)

S4 Fig Specificity of primer and probe sets for each HIV target RNA. cDNA amplicons were generated by RT-PCR with specific TaqMan primer and probe sets designed to target unspliced, multiply-spliced, Nef-encoding, Tat-encoding, and singly-spliced Env-encoding species of HIV RNA. These amplicons were used to generate plasmid standards of the HIV RNA species of interest, and their specificity determined by RT-qPCR. Results are shown for input amounts of 10^8^ copies per well.
(TIF)
