# Supplementary material for: HIV-1 latency is established preferentially in minimally activated and non-dividing cells during productive infection of primary CD4 T cells
Source: PLoS One. 2022 Jul 27;17(7):e0271674. doi: 10.1371/journal.pone.0271674 (PMC9328514; doi:10.1371/journal.pone.0271674)
Supplement: S1 Table — For the first three variables, results are given as the mean ± SD from 4 experiments, using cells from different donors. Cell restimulation data, averaged from 2 experiments. HIV DNA = total cell associated. Induction of productive virus replication (p24, pg/ml), at 7 days after a secondary stimulation was analyzed by ELISA. (DOCX) [file pone.0271674.s001.docx]

| **Day** | **% Cell Viability** | **% BrdU Incorporation** | **HIV DNA**  Copies/500ng | **Cell Restimulation**  p24(pg/mL) %Proliferation | |
| --- | --- | --- | --- | --- | --- |
| 1 | 88.0 + 2.3 | 0.2 + 0.3 | 26,405 | 135,220 | 85 |
| 2 | 86.3 + 5.1 | 0.0 + 0.1 | 15,012 | 51,780 | 83 |
| 3 | 75.8 + 7.5 | 0.0 + 0.1 | 15,349 | 49,711 | 82 |
