# Supplementary material for: HIV-1 latency is established preferentially in minimally activated and non-dividing cells during productive infection of primary CD4 T cells
Source: PLoS One. 2022 Jul 27;17(7):e0271674. doi: 10.1371/journal.pone.0271674 (PMC9328514; doi:10.1371/journal.pone.0271674)
Supplement: S2 Table — Primer and probe sequences used in the TaqMan qRT-PCR assay, for each of the five listed HIV transcript species. All probes were 5’ FAM and 3’ Black Hole Quencher conjugated. All targeted cDNA sequences were derived from the genomic sequence of the NL4-3 viral clone (Gen-Bank accession #M19921.1). (DOCX) [file pone.0271674.s002.docx]

| **Target** | **Primer** | **Sequence** |
| --- | --- | --- |
| Unspliced | Forward | AAAAGAGACCATCAATGAGGAAGC |
|  | Reverse | TGGTGCAATAGGCCCTGC |
|  | Probe | CAGAATGGGATAGATTGCATCCAGTGCA |
| Multiply-spliced | Forward | GCTCATCAGAACAGTCAGACTCATC |
|  | Reverse | TGTCGGGTCCCCTCGG |
|  | Probe | CTTCTCTATCAAAGCAACCCACCTCCCAAT |
| Env | Forward | CGACTGGAAGAAGCGGAGA |
|  | Reverse | ATTACTATGGACCACACAACTATTGC |
|  | Probe | CGACGAAGAGCTCATCAGAACAGTCAGACTC |
| Nef | Forward | GGCGACTGGAAGAAGCGG |
|  | Reverse | GGAGGTGGGTTGCTTTGATAGAG |
|  | Probe | AAGAGCTCATCAGAACAGTCAGACTCATCAAGCT |
| Tat | Forward | GGCGACTGAATTGGGTGTC |
|  | Reverse | TCTACTGGCTCCATTTCTTGCT |
|  | Probe | TCCTCTGTCGAGTAACGCCTATTC |
