# Supplementary figures and images for: HIV-1 latency is established preferentially in minimally activated and non-dividing cells during productive infection of primary CD4 T cells
Source: PLoS One. 2022 Jul 27;17(7):e0271674. doi: 10.1371/journal.pone.0271674 (PMC9328514; doi:10.1371/journal.pone.0271674)

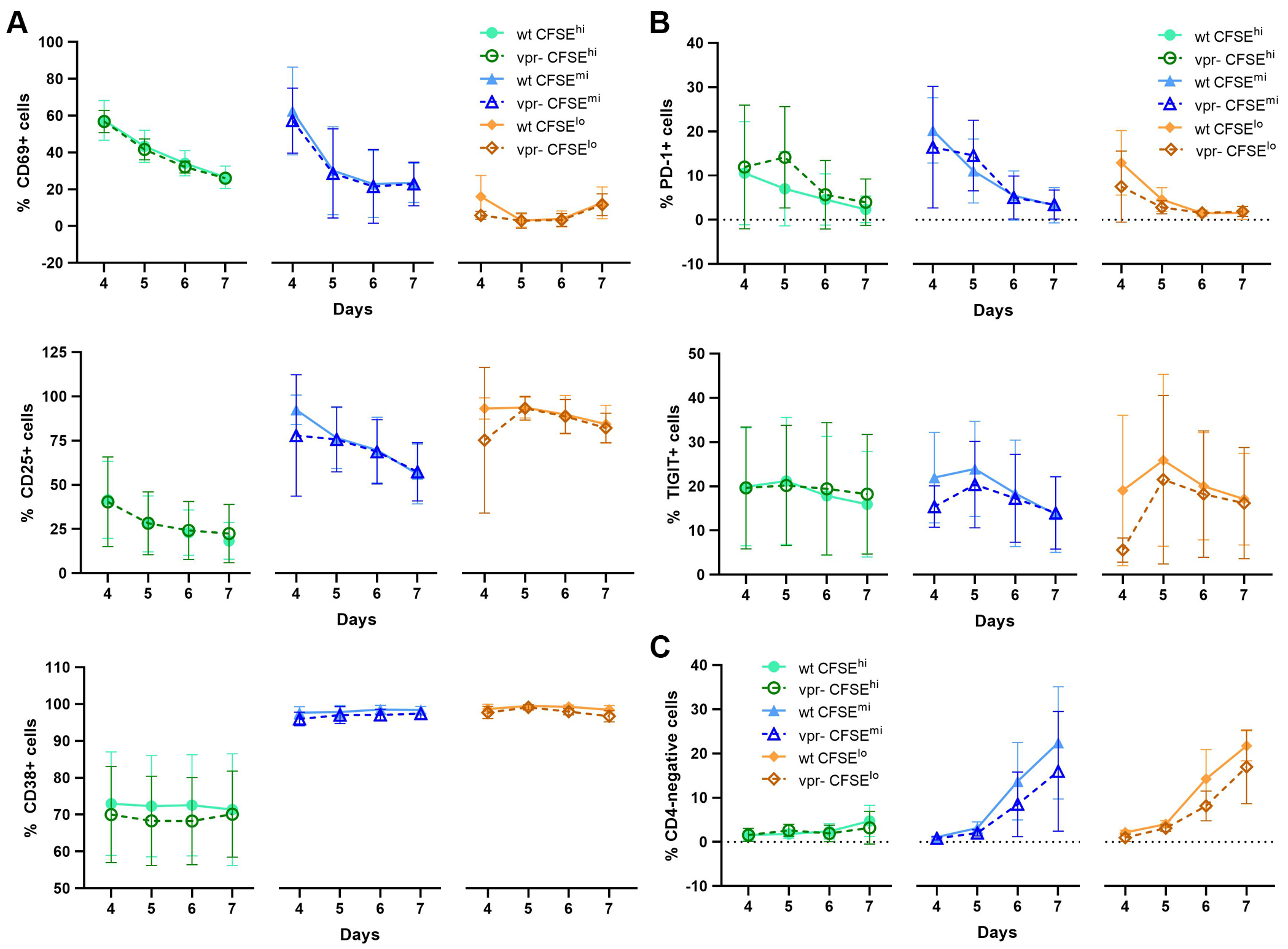

Supplement: S1 Fig — Aliquots of cells were stained with CFSE dye and infected in parallel with NL4-3 (wt) and vpr mutant (vpr-) virus (MOI 0.01 IU, 0.5 ml volume). Cells were activated on plates pre-coated with goat anti-mouse IgG plus anti-CD3 + anti-CD28 antibodies for 4 days. At this point, cells were removed from stimulation, resuspended in culture medium supplemented with IL-2 and IL-15, and transferred to new microculture plates. Samples were collected daily on days 4–7 for flow cytometry evaluation of cell surface antigen markers. Throughout the time course, wild type virus and vpr mutant were compared for expression of: (A) Activation markers (CD69, CD25, CD38); (B) Exhaustion-associated markers (PD-1 and TIGIT); (C) Surface expression of CD4 receptor. (TIF) [file pone.0271674.s003.tif]

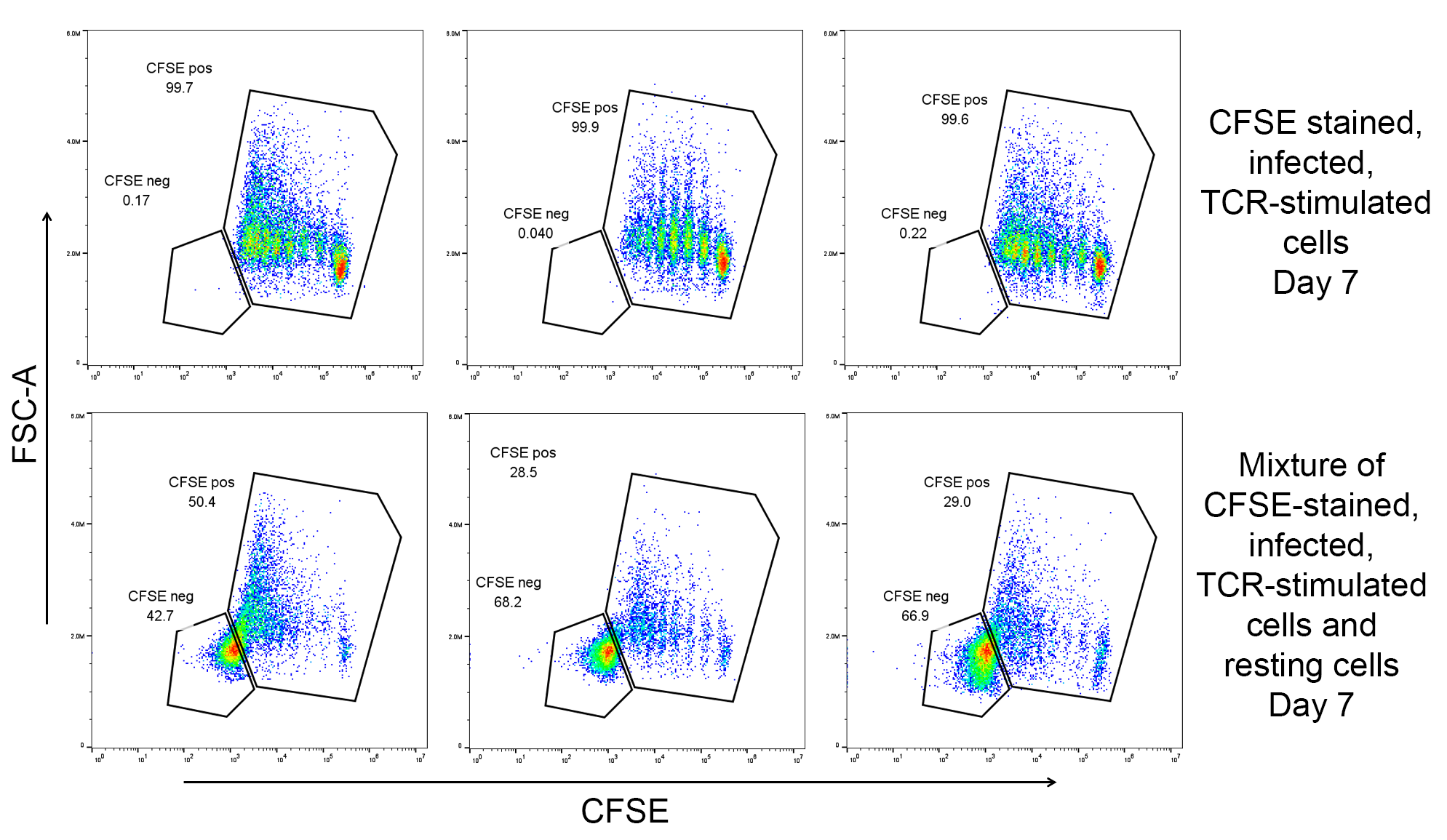

Supplement: S2 Fig — Gating regions were set to exclude any cells from the activated productively infected sample from the CFSE- population region. Despite potential loss of a small portion of resting CFSE- cells in some experiments (e.g. Donor 1), contamination with productively infected cells is very negligible to non-existent. (TIF) [file pone.0271674.s004.tif]

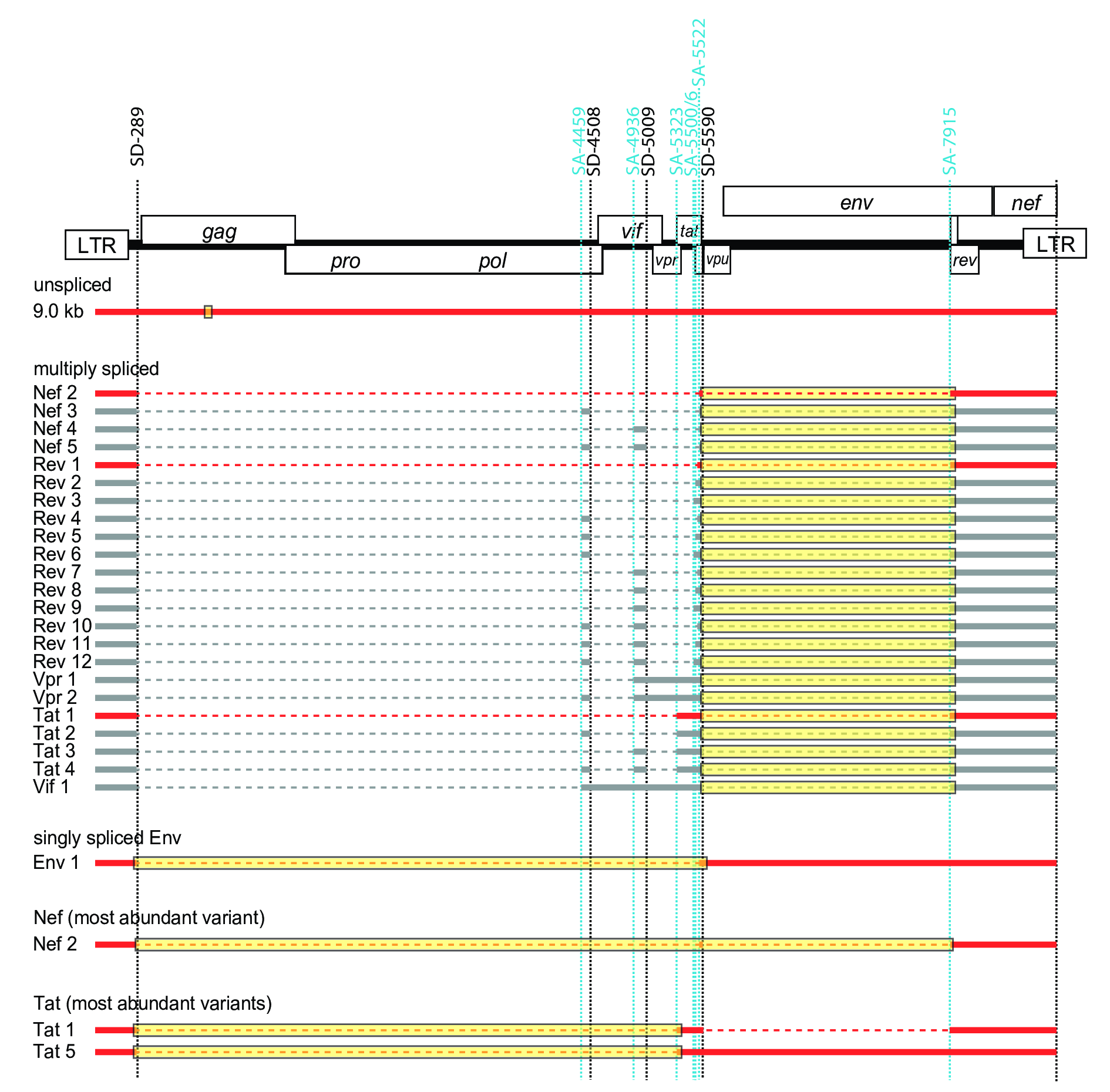

Supplement: S3 Fig — RNA specific Taqman amplicons are depicted by the yellow boxes. Primers or probes anneal across the splice donor (SD) and splice acceptor (SA) junctions. (TIF) [file pone.0271674.s005.tif]

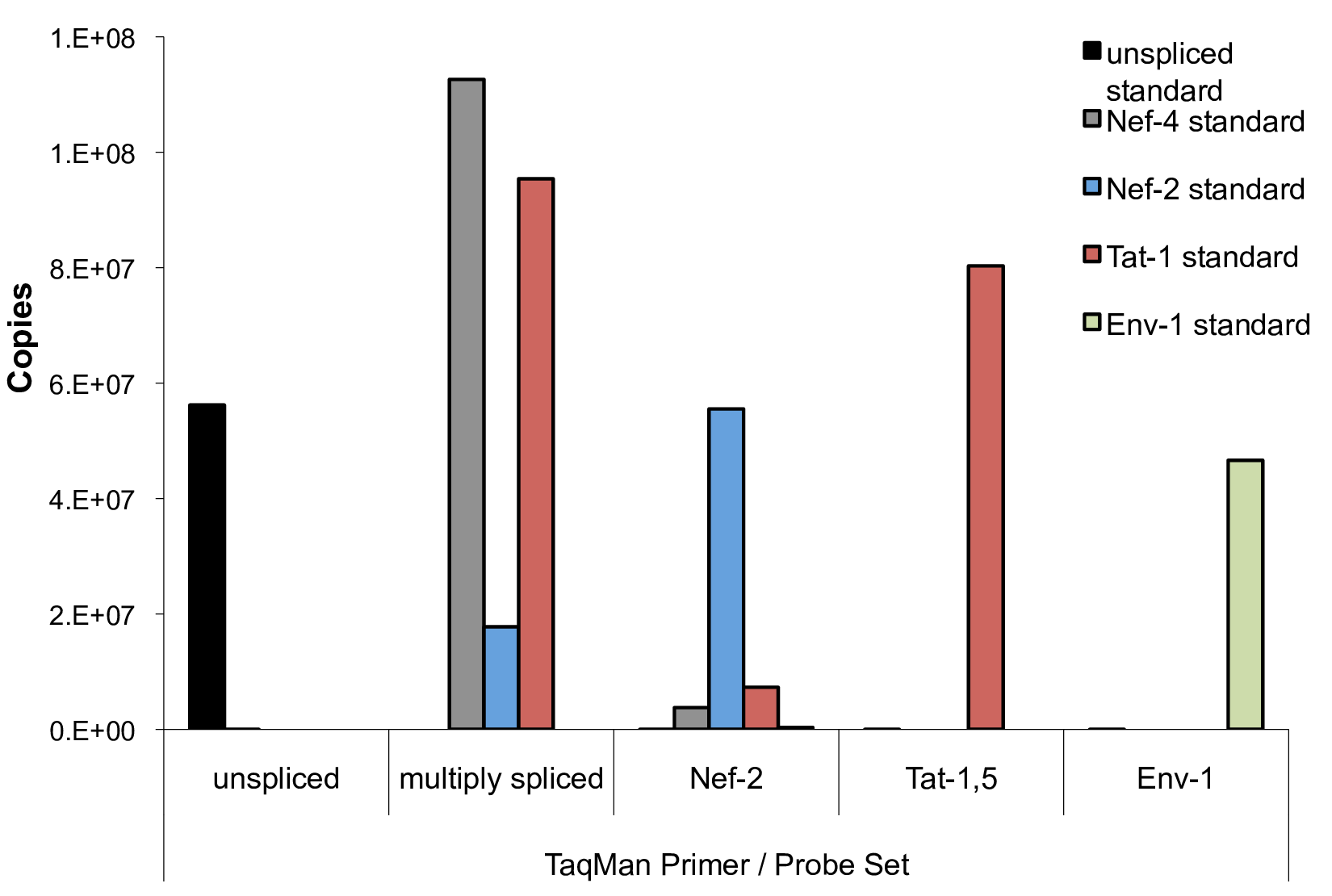

Supplement: S4 Fig — cDNA amplicons were generated by RT-PCR with specific TaqMan primer and probe sets designed to target unspliced, multiply-spliced, Nef-encoding, Tat-encoding, and singly-spliced Env-encoding species of HIV RNA. These amplicons were used to generate plasmid standards of the HIV RNA species of interest, and their specificity determined by RT-qPCR. Results are shown for input amounts of 108 copies per well. (TIF) [file pone.0271674.s006.tif]
